# Coronavirus genomes carry the signatures of their habitats

**DOI:** 10.1371/journal.pone.0244025

**Published:** 2020-12-22

**Authors:** Yulong Wei, Jordan R. Silke, Parisa Aris, Xuhua Xia

**Affiliations:** 1 Department of Biology, University of Ottawa, Ottawa, Ontario, Canada; 2 Ottawa Institute of Systems Biology, University of Ottawa, Ottawa, Ontario, Canada; University of Buffalo, UNITED STATES

## Abstract

Coronaviruses such as SARS-CoV-2 regularly infect host tissues that express antiviral proteins (AVPs) in abundance. Understanding how they evolve to adapt or evade host immune responses is important in the effort to control the spread of infection. Two AVPs that may shape viral genomes are the zinc finger antiviral protein (ZAP) and the apolipoprotein B mRNA editing enzyme-catalytic polypeptide-like 3 (APOBEC3). The former binds to CpG dinucleotides to facilitate the degradation of viral transcripts while the latter frequently deaminates C into U residues which could generate notable viral sequence variations. We tested the hypothesis that both APOBEC3 and ZAP impose selective pressures that shape the genome of an infecting coronavirus. Our investigation considered a comprehensive number of publicly available genomes for seven coronaviruses (SARS-CoV-2, SARS-CoV, and MERS infecting *Homo sapiens*, Bovine CoV infecting *Bos taurus*, MHV infecting *Mus musculus*, HEV infecting *Sus scrofa*, and CRCoV infecting *Canis lupus familiaris*). We show that coronaviruses that regularly infect tissues with abundant AVPs have CpG-deficient and U-rich genomes; whereas those that do not infect tissues with abundant AVPs do not share these sequence hallmarks. Among the coronaviruses surveyed herein, CpG is most deficient in SARS-CoV-2 and a temporal analysis showed a marked increase in C to U mutations over four months of SARS-CoV-2 genome evolution. Furthermore, the preferred motifs in which these C to U mutations occur are the same as those subjected to APOBEC3 editing in HIV-1. These results suggest that both ZAP and APOBEC3 shape the SARS-CoV-2 genome: ZAP imposes a strong CpG avoidance, and APOBEC3 constantly edits C to U. Evolutionary pressures exerted by host immune systems onto viral genomes may motivate novel strategies for SARS-CoV-2 vaccine development.

## Introduction

The COVID-19 pandemic is a serious global health emergency. Understanding how coronaviruses adapt or evade tissue-specific host immune responses is important in the effort to control the spread of infection and to facilitate vaccine-development strategies. As obligate parasites, coronaviruses evolve in mammalian hosts and carry genomic signatures shaped by their host-specific environments. For instance, SARS-CoV-2 regularly infects bronchiolar and type II alveolar epithelial cells in the lungs [1] and enterocytes in the small intestines [2]. At the tissue level, hosts provide different cellular environments with varying levels of antiviral activity. Two antiviral proteins (AVPs) that may contribute to the modification of viral genomes are the zinc finger antiviral protein (ZAP, gene name ZC3HAV1 in mammals) and the apolipoprotein B mRNA-editing enzyme-catalytic polypeptide-like 3 (APOBEC3) protein, both of which exhibit tissue-specific expressions [3].

### ZAP is endogenously expressed in tissues and relies on motif-specific transcript targeting to mediate antiviral activity

ZAP is a key component in the mammalian interferon-mediated immune response that specifically targets CpG dinucleotides in viral RNA genomes [4] to inhibit viral replication and signal for viral genome degradation [4–7]. ZAP acts against retroviruses such as HIV-1 [5, 8], and single-stranded RNA viruses such as Ecovirus 7 [9], Zika virus [10], and Influenza virus [11]. It follows that cytoplasmic ZAP activity should impose a strong CpG avoidance in RNA viruses that infect tissues abundant in ZAP. For instance, while HIV-1 infects lymph organs where ZAP is abundant [3], its genome is also strongly CpG-deficient, and the viral fitness of HIV-1 diminishes as its genomic CpG content increases within a sample of patients [12]. Indeed, many single-stranded RNA viruses exhibit strong CpG deficiency [7, 11, 13–15], but selection for CpG deficiency disappears in ZAP-deficient cells [7]. Furthermore, ZAP may prefer to target CG dinucleotides in specific CG-rich ssRNA contexts. Based on an *in vitro* crystal structure study that examined the binding affinity between the mouse ZAP zinc-finger motif and a variety of CG-rich ssRNA sequences [16], the mouse ZAP-preferred motifs were determined to be CN_X_GNCG, where N_X_ is a spacer sequence of length 4nt to 8nt. However, to date, a human ZAP-preferred consensus motif remains to be determined [4].

Recent studies have shown strong CpG deficiency in the SARS-CoV-2 genome in comparison to SARS-CoV and MERS-CoV, and they suggested that SARS-CoV-2 is adapted to evade the ZAP antiviral defense [17, 18]. Indeed, endogenous ZAP activity has been shown to restrict SARS-CoV-2 replication in human lung cell lines as they express ZAP in abundance [17].

### APOBEC3 is highly expressed in immune cells and is also detected in tissue-specific cell lines

Aside from ZAP, the APOBEC3 cytidine deaminase enzymes have garnered substantial attention for their role in the antiviral immune response [19, 20]. Unlike ZAP, APOBEC3 enzymes are mainly expressed in hematopoietic cell populations, including T cells, B cells, and myeloid cells [21]. Consequently, APOBEC3 enzymes are highly expressed in lymphoid organs including the thymus, spleen, and lymph nodes [21, 22]; however, APOBEC3 expression is not confined to lymphoid organs. Two studies have detected APOBEC3-encoding mRNAs from total RNA of non-lymphoid tissues [21, 22], and they suggested that APOBEC3 enzymes are variably expressed in these tissues due to differing lymphocyte contents. For example, a considerable number of APOBEC3-expressing macrophages reside in the lung alveoli, and expectedly, both Koning et al. [21] and Refsland et al. [22] found the highest levels of APOBEC3 expression in lung tissues among non-lymphoid tissues.

Additionally, among tested APOBEC3 RNAs, A3A, A3B, A3F, and A3G, but not A3H, were detected in the human mammary epithelial cells [23], and transcripts of both A3G and A3F were detected in human lung epithelial cells [24]. In particular, A3G transcripts were upregulated in response to viral infection in human lung epithelial cells. Although it remains unclear whether APOBEC3 expression in tissue cells plays a role in restricting viral infections, APOBEC3 expression has been observed to vary at the tissue level [21, 22].

### Catalytic activity of APOBEC3 is induced in response to cell stress and the enzymes prefer specific motif contexts

APOBEC3 enzymes have negligible catalytic activity prior to infection, but expressions of associated mRNAs are induced in response to cell stresses including hypoxia, cell crowding, and presence of interferon-α [21, 25, 26]. Through a mechanism largely derived from HIV-1 studies, APOBEC3 enzymes have been prominently reported to restrict viral infectivity [20, 27–30] by editing C to T at the viral genomes, and HIV-1 avoids this deleterious effect by expressing Vif to target and degrade APOBEC3 enzymes [31, 32].

Additionally, C to U RNA-editing has been demonstrated in lymphocytes, macrophages, monocytes, and natural killer cells by both A3A and A3G in response to hypoxia and interferons [25, 26, 33], and recent studies propose that APOBEC3 enzymes may act directly to edit single-stranded RNA coronaviruses [34–36]. This notion is supported by lines of evidence showing that A3C, A3F, and A3H may inhibit HCoV-NL63 coronavirus infection in humans [37]. Indeed, many C to U mutations have been detected in SARS-CoV-2 genomes [34–36, 38], and if RNA-editing by APOBEC3 is involved, then this immune response could potentially restrict SARS-CoV-2 because coronaviruses do not encode a Vif analogue to degrade APOBEC3 enzymes.

Nonetheless, APOBEC3 enzymes prefer to edit C in specific motif contexts. For instance, in HIV-1 [39, 40], MLV [41–43], and SIV [44], A3G tends to deaminate C mostly in the context of 5’ CC (underlined is site subjected to C to T editing), whereas all other APOBEC3 paralogues deaminate C in the context of 5’ TC [40, 45–47]. In HIV, these edits cause 5’ GG to 5’ AG and 5’ GA to 5’ AA hypermutations on the positive DNA strand [39, 40] to potentially disrupt protein function [48, 49]. However, not all 5’ TC and 5’ CC are deaminated with equal efficiency because the identities of the -2 and +1 nucleotides flanking the 5’NC are important in APOBEC3 target selection [41, 50–53].

### APOBEC3 editing activity is influenced by substrates’ higher-order structure

In addition to motif preference, the structural configuration of the substrates bound to the APOBEC3 zinc center may also influence APOBEC3 editing activity [54, 55]. Adding to this complexity, APOBEC3-mediated editing studies have reached dissimilar conclusions as to the optimal secondary structure of the 5’ TC target. For instance, a large number of A3A and A3G RNA editing substrates were predicted to form a loop structure in innate immune cells and HEK293T cells [25, 33]. In a further mutagenesis study on three A3A editing RNA substrates, *SDHB*, *APP*, and *TMEM109*, and on one A3G editing RNA substrate *PRPSAP2*, Sharma and Baysal [56] found that both A3A and A3G enzymes highly preferred to edit the respective 5’ TC and 5’ CC targets that resided within a 4nt-loop in a stem-loop structure, with C located at the 3’ end of the loop followed by a +1G located at the 5’ end of the stem. In this structural context, changing the substrate at the -1 and +1 nucleotides greatly reduced A3A and A3G editing activities [56]. Nonetheless, a limitation of the study is that only four APOBEC3 editing substrates were examined and all were in the context of 5’ N(C/T)CG.

Another study [57] showed that A3G could also efficiently edit 5’ ACCA, 5’ CCCC, and 5’ TCCT, but not 5’ GCCG, in ssDNA oligonucleotides when these targets were in an open (unstructured) configuration. Furthermore, A3G poorly edits 5’ ACCA and 5’ CCCC targets when they are located in short loops (<7 nt and <6 nt, respectively). Summarily, A3G prefers to edit 5’ CC targets within a loop region but only in the context of 5’ NCCG.

A third study [52] analyzed the *in vitro* editing activities of all seven APOBEC3 enzymes on ssDNA oligonucleotides embedding 5’ NTCN motifs, where the 5’ TC targets were located within loop, stem, or open structures. McDaniel et al. [52] found that the editing activities of all APOBEC3 enzymes, except A3F and A3H, were the highest when 5’ TC in the context of 5’ GTCG was located within a loop region. However, all seven APOBEC3 enzymes also had the lowest editing activities at 5’ GTCG in comparison to other 5’ NTCN motifs, and the editing activities of all APOBEC3 enzymes were the highest when 5’ ATCA, 5’ GTCA, 5’ CTCA, and 5’ CTCT were located in an open structure. In general, APOBEC3-edited 5’ (C/T)C targets in the context of 5’ N(C/T)CG prefer a loop region, but 5’ TC targets in the context of 5’ NTC(A/T) prefer an open structure.

### Host immune responses exert selective pressures that shape the genomic composition of tissue-specific coronaviruses

The above observations allow for the formation of the hypothesis that APOBEC3 and ZAP exert selective pressure on coronavirus genomes. To test this, a variety of mammalian hosts and tissues should be considered because there may be both species-specific and tissue-specific differences in ZAP and APOBEC3 productions. If the short-lived mice should produce less ZAP and APOBEC3 than long-living mammals, then the mouse hepatitis virus (MHV) should also experience little selection targeting CpG by ZAP and C to U editing by APOBEC3 in comparison to other mammalian-specific coronaviruses. Moreover, coronaviruses regularly infect organ tissues exposed to the external environments such as the respiratory and digestive systems [58, 59]. We expect that if a coronavirus regularly infects host tissues that are abundant in ZAP, then its genome should display CpG deficiency in CG-rich motifs such as CN_X_GNCG, to elude ZAP-mediated immune response. If, in addition, the regularly infected tissue is abundant in APOBEC3, then the viral genome should trend prevalently towards increased C to U mutations in the context of APOBEC3-preferred motifs. Conversely, if a species-specific coronavirus regularly infects host tissues that are deficient in either APOBEC3 or ZAP, there will be either no strong CpG deficiency or elevated U and decreased C contents, as these selective pressures will be weak.

Our investigation considered a comprehensive number of publicly available genomes for seven coronaviruses (the Betacoronaviruses: SARS-CoV-2, SARS-CoV, MERS, Bovine CoV, mouse hepatitis virus [MHV], and porcine hepatitis E virus [HEV], and the Alphacoronavirus canine respiratory coronavirus [CRCoV]) as well as studies with tissue-level *ZAP* and *APOBEC3* mRNA expressions in the five host species (human, cattle, dog, mice, and pig). We found that all surveyed coronaviruses, except MHV, regularly infect host tissues with high *ZAP* and *APOBEC3* mRNA expressions. Expectedly, all surveyed coronavirus genomes except MHV are strongly CpG deficient. In addition, deficiency of CpG was detected in the context of ZAP-preferred motifs in SARS-CoV-2. Furthermore, a temporal and geographical analysis for single nucleotide polymorphisms (SNPs) in local SARS-CoV-2 regions showed that the occurrence of C to U mutations was strikingly more prevalent than other SNPs. The preferred motif and structural contexts of 5’ UC to 5’ UU mutations were consistent with those favorably edited by APOBEC3 enzymes, but 5’ CC to 5’ CU mutations were weakly explained by APOBEC3G editing preference. The genome compositions of viruses are subjected to adaptation when the virus regularly infects tissues expressing ZAP and APOBEC3 in abundance, but not when a virus infects tissues that lowly express these AVPs.

## Materials and methods

### Retrieving and processing the *APOBEC3* and *ZAP* genes and their tissue level mRNA expressions in five mammalian species

The NCBI Nucleotide Database was queried for “APOBEC3” and “ZC3HAV1” as gene names, and “*Homo sapiens*”, “*Bos taurus*”, “*Canis lupus familiaris*”, “*Mus musculus*”, and “*Sus scrofa”* as species, and protein coding sequences of APOBEC3 and ZC3HAV1 isoforms were extracted in FASTA format along with their Ensembl Accession IDs.

To compare mRNA expressions of *APOBEC3* and *ZC3HAV1* (ZAP) among tissues, we retrieved publicly available RNA Sequencing and Microarray studies that each sampled total RNA in at least ten mammalian tissues (see S1 File). The five mammalian species that have extensive tissue-specific mRNA expressions are *Homo sapiens* (human), *Bos Taurus* (cattle), *Canis lupus familiaris* (dog), *Mus musculus* (mice), and *Sus scrofa* (pig). For *Homo sapiens*, tissue-level mRNA expressions were retrieved in averaged FPKM values from all 171 RNA-Seq datasets in BioProject PRJEB4337 [3], 48 RNA-Seq datasets in BioProject PRJEB2445, 20 RNA-Seq datasets in BioProject PRJNA280600 [60], and in median TPM values from all RNA-Seq datasets available in the GTEx Portal [61]. For *Mus musculus*, tissue-level mRNA expressions were retrieved in averaged FPKM values from all 741 RNA-Seq datasets in BioProject PRJNA66167 (mouse ENCODE consortium) [62] and in average TPM values from all 79 RNA-Seq datasets in BioProject PRJNA516470 [63]. For *Sus scrofa*, tissue-level mRNA expressions were retrieved in averaged FPKM values from TISSUE 2.0 integrated datasets [64]. For *Canis lupus familiaris*, tissue-level mRNA expressions were retrieved in averaged fluorescence intensity units (FIU) from all 39 microarray datasets in BioProject PRJNA124245 [65], and in averaged TPM values from all 75 RNA-Seq datasets in BioProject PRJNA516470 [63]. Lastly, for *Bos taurus*, tissue-level mRNA expressions were retrieved in averaged FPKM values from 42 RNA-Seq datasets in the Bovine Genome Database [66]. All selected studies have considered total RNA at the tissue level in healthy individuals, but they do not report cell-specific mRNA expressions within most tissues (e.g., lung, liver, small intestine).

Given that mRNA expressions were extracted were from multiple independent sources (some reporting FPKM and others TPM) and thus not directly comparable between studies, we calculated the relative mRNA expression levels of APOBEC3 and ZAP isoforms among tissues in each independent source. Specifically, we calculated the proportion of mRNA expression (PME) as:

$$PME=\frac{\text{mRNA}\;\text{expression}\;\text{value}\;\text{in}\;\text{a}\;\text{specific}\;\text{tissue}}{\text{summed}\;\text{mRNA}\;\text{expression}\;\text{values}\;\text{in}\;\text{all}\;\text{tissues}}$$ (1)

To show that PME determines the relative mRNA expressions of a gene among tissues, we calculated the PME values for all 13 human genes that were determined to have the highest mRNA expressions in the lungs (marked as Tissue enriched) by The Human Protein Atlas database [67] (https://www.proteinatlas.org/humanproteome/tissue/lungs). They are *SFTPC*, *SFTPA2*, *SFTPA1*, *SCGB1A1*, *SFTPB*, *AGER*, *SCGB3A2*, *SFTA2*, *CACNA2D2*, *LAMP3*, *SFTPD*, *HTR3C*, *RTKN2*. If PME works as intended, then the PME values of these 13 genes should be high in the lung tissue in comparison to 52 other tissues reported in the GTEx database [61]. As expected, we found that 12 out of these 13 genes have the highest PME values in the lungs, but *CACNA2D2* has the highest PME value in the cerebellum and second highest PME values in the lungs (see S1 File). This is not unexpected because based on the BRAIN ATLAS [67], the mRNA expression of *CACNA2D2* is also enhanced in the cerebellum.

PME values were calculated from averaged TPM values in 24 human tissues using all RNA-Seq datasets available in the GTEx Portal [61], from averaged FPKM values in 26 cattle tissues using the Bovine Genome Database [66], from averaged FPKM values in 33 pig tissues using TISSUE 2.0 integrated datasets [64], from averaged FPKM values in 17 mice tissues using all 741 RNA-Seq datasets in mouse ENCODE consortium [62], from averaged FPKM values in 12 mice tissues using 79 RNA-Seq datasets in BioProject PRJNA516470 [63], and from averaged fluorescence intensity units in 10 dog tissues using all 39 microarray datasets in BioProject PRJNA124245 [65]. For each AVP isoform, tissue-specific PMEs were designated as high if they are greater than averaged PME and low if they are less than averaged PME (see S1 File).

### Processing and quantifying transcriptomic data from chimeric human lung-only mice to obtain AVP mRNA expressions in control vs. SARS-CoV-2-infected human lung epithelial cells

Transcriptomic data associated with a study exploring SARS-CoV-2 infection in chimeric human lung-only mice (LoM) (GSE155286) were retrieved from NCBI’s Sequence Read Archive (SRA) database and a summary of the data collected is detailed in Table 1.

**Table 1:** Summary of the RNA-seq dataset used to quantify genes of interest across SARS-CoV-2 infection states.

Series	Infection state	Sample	Experiment	Runs
GSE155286	Control	GSM4698496	SRX8839384	SRR12339593
				SRR12339594
		GSM4698497	SRX8839385	SRR12339595
				SRR12339596
	2 days after	GSM4698487	SRX8839375	SRR12339575
				SRR12339576
		GSM4698488	SRX8839376	SRR12339577
				SRR12339578
	6 days after	GSM4698490	SRX8839378	SRR12339581
				SRR12339582
		GSM4698491	SRX8839379	SRR12339583
				SRR12339584
	14 days after	GSM4698493	SRX8839381	SRR12339587
				SRR12339588
		GSM4698494	SRX8839382	SRR12339589
				SRR12339590

Column 2 describes the infection states of the human lung epithelial cells, with “Control” = samples collected prior to SARS-CoV-2 infection, and “# days after” = samples collected at # days after SARS-CoV-2 infection

The data were first partitioned into gzipped forward and reverse read fastq files using fastq-dump from the NCBI SRA toolkit (version 2.10.8). The resulting fastq.gz files were trimmed for Illumina TruSeq3 adapters and reads averaging a phred quality score < 20 were discarded using trimmomatic version 0.39 [68]. All surviving pairs from preprocessing were carried forward to quantification using kallisto (version 0.46.1) [69].

An index file for the human transcriptome was generated from the Ensembl FASTA reference file “Homo_sapiens.GRCh38.cdna.all.fa” containing all human cDNAs with Ensembl transcript IDs [70] using kallisto’s index function. The resulting index was used to quantify transcript abundances using kallisto for each experiment detailed in Table 1, and 1000 bootstrap samples were computed for each experiment to act as a proxy for technical replicates during subsequent analysis using sleuth (version 0.30) [71].

The kallisto outputs, including bootstrapped values from the previous step, were processed using the sleuth R package. Ensembl transcript IDs were associated with their Ensembl Gene ID and gene name using the biomaRt R package and a sleuth object was prepared using the Ensembl gene ID for aggregation. The sleuth object was then fitted with two models: a full model (alternative) that assumes transcript abundance varies based on the time after SARS-CoV-2 infection, and a reduced model (null) assuming that transcript abundance varies between samples. The two models were compared with the likelihood-ratio test, and the resulting transcript level p-values were then aggregated based on their associated Ensembl gene ID using Lancaster’s method, which assigns weights based on transcript abundance. A Benjamini-Hochberg false discovery rate correction [72] was then applied to the weighted p-values to account for multiple comparisons [73]. AVP genes were then differentially assessed at the level of their corresponding transcripts and comparisons were drawn from heat maps using natural log transformed TPM values with a 0.5 offset generated by sleuth. Only transcripts of interest with an Ensembl Biotype of “Protein coding” that demonstrated variations in expression levels were considered in subsequent analyses. All significantly differentially expressed genes between control and infected samples, with false discovery rate q < 0.05, are listed in S1 File.

### Determining the regular habitats of coronaviruses infecting five mammalian species

Host tissues that are infected by SARS-CoV-2, SARS-CoV, and MERS in humans, Bovine CoV in cattle, CRCoV in dogs, MHV in mice, and HEV in pigs were identified through an exhaustive large-scale manual search for experimental evidence-based primary source studies published up until June 5, 2020. Only studies that showed results from clinical course, autopsy, and experimental infections were considered, but cross-host studies were excluded. In total, tissue infections were determined from 25 SARS-CoV studies, 11 SARS-CoV-2 studies, eight MERS studies, 15 mouse hepatitis virus (MHV) studies, nine porcine hepatitis E virus (HEV) studies, 18 canine respiratory coronavirus (CRCoV) studies, and ten bovine coronavirus (Bovine CoV) studies (see references in S1 File). Next, the regular tissue habitats of viruses were determined based on commonness of viral detection in host tissues when all studies were considered. For example, among the 25 SARS-CoV-2 studies collected, some tissue infections (e.g., lungs and intestines) are recorded in many studies while other tissue infections are rarely recorded (e.g., stomach). To score the commonness of SARS-CoV-2 infection in a tissue, in the lungs for instance, we calculated commonness of detection (COD) as:

$$COD=\frac{\text{number}\;\text{of}\;\text{times}\;\text{lungs}\;\text{infection}\;\text{is}\;\text{recorded}\;\text{in}\;\text{all}\;\text{studies}\;\text{considered}}{\text{total}\;\text{number}\;\text{of}\;\text{recorded}\;\text{infections}\;\text{in}\;\text{all}\;\text{tissues}\;\text{in}\;\text{all}\;\text{studies}\;\text{considered}}$$ (2)

Note that the COD measurement should not be used to make specific comparisons to rank most to least regularly infected tissues, because manually curated study size biases COD measurements. However, COD does tell us which tissues were commonly infected by a virus. For example, among the 25 SARS-CoV-2 studies collected, viral detection was reported in the lungs in nine studies, the intestines in eight studies, the liver in four studies, the heart in three studies, the kidney in three studies, and the stomach in one study (S1 File). The COD values for the lungs and the intestines are therefore the highest. Hence, the lungs and the intestines are surely regular habitats of SARS-CoV-2. Similarly, we determined the regular habitat for SARS-CoV (human lungs), MERS (human lungs), MHV (mice brain), HEV (pig liver), CRCoV (dog intestines and lungs), and Bovine CoV (cattle intestines and lungs). These regular habitats have COD values higher than twice that of any other tissue, except dog lungs for CRCoV, whose COD value was at least 1.5 times that of other tissues.

### Retrieving and processing the genomes of coronaviruses infecting five mammalian species

The genome, Accession ID, and Sample Collection Date of 28475 SARS-CoV-2 strains were retrieved from the China National Center for Bioinformation (CNCB) (https://bigd.big.ac.cn/ncov/variation/statistics?lang=en, last accessed May 16, 2020), among which 2666 strains were selected because they were annotated as having complete genome sequences and high sequencing quality. Additionally, the complete genomic sequences of 403 MERS strains, 134 SARS-CoV strains, 20 Bovine CoV strains, two CRCoV strains, 26 MHV strains, and ten HEV strains were downloaded from the National Center for Biotechnology Information (NCBI) Nucleotide Database (https://www.ncbi.nlm.nih.gov/) (see S2 File).

We computed the nucleotide and di-nucleotide frequencies in each viral genome. Among strains, some have long poly-A tails that are missing in others. Some also have a longer 5’ untranslated region (5’ UTR) than others. To make a fair comparison between strains, genomes were first aligned with MAFFT version 7 [74], with the slow but accurate G-INS-1 option for 134 SARS-CoV, 20 Bovine CoV, two CRCoV, 26 MHV, and ten HEV strains, and with the fast FFT-NS-2 option for large alignments for 2666 SARS-CoV-2 and 403 MERS strains. Next, using DAMBE version 7 [75], the 5’ UTR sequences were trimmed away until the first fully conserved nucleotide position, and the 3’ UTR sequences were trimmed out up to the last fully conserved nucleotide position. Then, gaps were removed from each trimmed genome, and the global nucleotide and dinucleotide frequencies were computed in DAMBE under “Seq. Analysis|Nucelotide & di-nuc Frequency” (see S2 File). Additionally, nucleotide and di-nucleotide frequencies were similarly computed for whole, untrimmed, genomes (see S3 File). Finally, the conventional index of CpG deficiency (I_CpG_) [76, 77] was calculated, using the formula below:

$$I_{CpG}=\frac{P_{CG}}{P_{C}P_{G}}$$ (3)

Where P_CG_ is the proportion of CG dinucleotides when all dinucleotide frequencies were considered, and P_C_ and P_G_ are proportions of C and G nucleotides, respectively. The index is expected to be proximal to 1 when CpG is not deficient or in excess, smaller than 1 if CpG is deficient and greater than 1 if CpG is in excess.

### Determining the temporal and geographical patterns of SNPs in SARS-CoV-2 genomes

Among the 2666 SARS-CoV-2 genomes from CNCB (database last accessed on May 16, 2020), we randomly selected one genome at each unique collection date, inclusively between December 31, 2019 (Wuhan-Hu-1, first isolate) and May 6, 2020 (mink/NED/NB04), among those that have complete records of local region annotations and nucleotide sequences in NCBI (see S4 File). A total of 99 strains were retrieved across 127 days since SARS-CoV-2 (including strain Wuhan-Hu-1, MN908947) was first sequenced. For each of these 99 strains, the nucleotide sequence of 12 out of 13 viral regions (5’ UTR, ORF1ab, S, ORF3, E, M, ORF6, ORF7a, ORF8, N, ORF10, and 3’ UTR) were extracted from DAMBE in FASTA format, MAFFT aligned with the slow but accurate G-INS-1 option, and local nucleotide and dinucleotide frequencies were computed for each region (see S5 File). ORF7b was omitted from the analysis because it was not annotated in 30 out of 99 strains, including the reference genome Wuhan-Hu-1 (MN908947).

To determine the nucleotide mutation patterns over time at each viral region, each aligned sequence was grouped into one of six time ranges, and the time range within each group was determined as the number of days passed since the reference strain (Wuhan-Hu-1, 2019-12-31). Note that the number of days between time intervals is unequal, because strains were grouped based on roughly equal sample size and not by equal number of days. Then, sequences within time groups were pair-wise assessed for single nucleotide polymorphisms (SNPs) using DAMBE’s “Seq. Analysis|Nucleotide substitution pattern” with reference genome = Wuhan-Hu-1 (MN908947) and Default genetic distance = F84, and the sum of SNPs within each group was calculated (see S4 File).

To control for any confounding effects imposed by mutations that could arise in specific geographic areas, we repeated the above analysis for all high quality and complete genomes in a country-specific manner. Only three countries have sequenced large numbers of strains with unique collection dates, leading us to consider 80 strains from the United States, 39 strains from Australia, and 34 strains from China (see S4 File). Note that because sample collection dates vary from one country to another, the time intervals will differ among geographical locations. In addition, within a geographical location, the sample sizes and time intervals may differ slightly among viral regions because not all strains have complete annotations for every viral region. For example, all 34 strains from China have an annotated E region, but two out of the 34 strains are missing an annotation for ORF8. Nucleotide mutations in these strains were traced relative to the reference genome being the oldest available strain in each country: MN908947 in China (2019-12-31), MN985325 in the US (2020-01-19), and MT450920 in Australia (2020-01-25). The statistical significance of C to U mutations relative to all other mutations was established using the non-parametric Wilcoxon rank-sum test with continuity correction.

### Sequence context and structural analyses of C to U mutations in the SARS-CoV-2 genome

The count, location, and identity of all non-synonymous SNPs were determined for each MAFFT aligned protein coding region (e.g., ORF1ab, see S6 File) using DAMBE’s “Seq. analysis|Codon substitution pattern, reference = Wuhan-Hu-1, MN908947”. Next, the count, identity, and location of all SNPs at each viral region were determined using DAMBE’s “Seq. analysis|Site-specific Nuc. Freq.”. This output was then compared with the output that contains non-synonymous substitutions to obtain the count, identity, and location of all synonymous substitutions. Similarly, the count, identity, and location of all non-coding SNPs in the two non-protein coding regions (5’ UTR and 3’ UTR) were determined using DAMBE’s “Seq. analysis|Site-specific Nuc. Freq.”.

The above outputs were further processed to determine the unique locations to obtain site-specific C to U mutations in each viral region. These outputs were used to determine the identities of the flanking nucleotides for all site-specific C to U mutations to generate the 5’ NC, 5’ NNC, and 5’ NNCN motif contexts (underlined are the C to U mutation sites, and N is any nucleotide). Next, the total numbers of 5’ NC, 5’ NNC, and 5’ NNCN motifs in the Wuhan-Hu-1 genome were determined using DAMBE’s “Sequences|Extract motif context”. Finally, these values were used to calculate the odds-ratio for each motif: the observed proportion of motifs with C to U mutations (e.g., number of 5’ AC with C to U mutations divided by total number of 5’ AC dinucleotides in Whuan-Hu-1 genome = 36/2023) divided by the expected proportion of C to U mutation (total number of C to U mutations divided by total number of C in Wuhan-Hu-1 genome = 98/5492). For example, the odds-ratio of 5’ AC is (36/2023)/(98/5492) = 0.997.

Next, for putatively edited C containing substrates on the Wuhan-Hu-1 genome, a 5 nt motif NNCNN was extended by 8 nt on either side to obtain a 21 nt sequence. To obtain the folding energy of the 21 nt sequence and obtain the secondary structure of the 5’NNCN motif, we used Minimum Folding Energy (MFE,—kcal/mol) via the Vienna RNA Folding Library [78], with the following options: no lonely pairs, Temperature = 37°C (S6 File).

## Results

### All surveyed coronaviruses except MHV regularly infect host tissues that highly express both ZAP and APOBEC3

**Fig 1:**
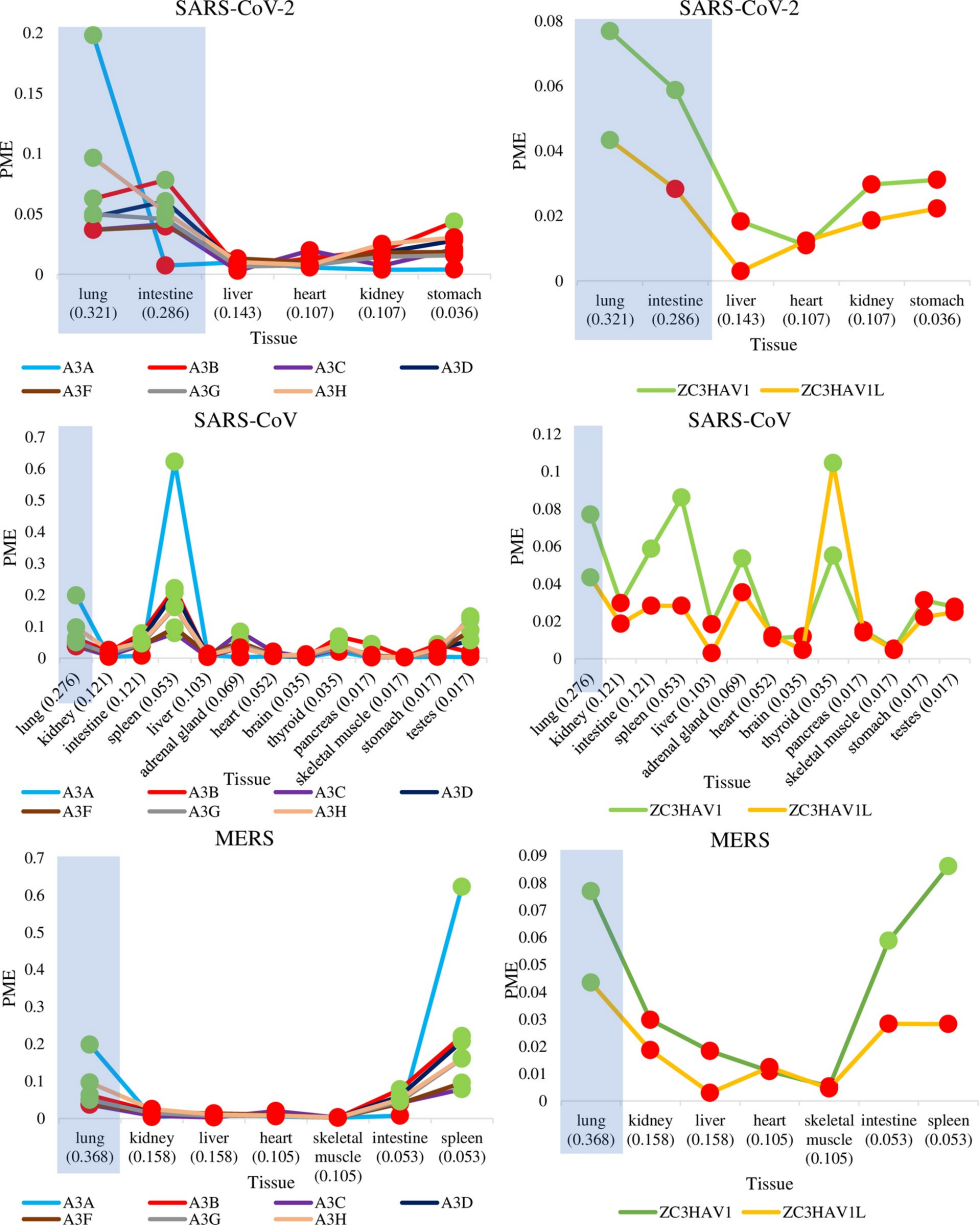
Tissues that are regularly infected by SARS-CoV-2, SARS-CoV, and MERS also have high mRNA expressions of *APOBEC3* and *ZAP* AVPs. The lines show the relative mRNA expressions in PME, for each *APOBEC3* and *ZAP* isoform, among tissues having records of SARS-CoV-2, SARS-CoV, and MERS infections. Dots highlighted in green and red are PME values that are greater and lower than the averaged PME values, respectively. These PME values were calculated based on averaged mRNA FPKMs retrieved from the GTEx Portal [61]. For each tissue, the commonness of viral detection (COD) score is appended in brackets next to tissue name. Shaded in light blue-gray are tissues that were determined to be regularly infected by the coronavirus (based on highest COD scores).

We determined which human tissues are regularly infected by coronaviruses and whether these tissues express ZAP and APOBEC3 in abundance. S1 Fig shows the tissue-specific mRNA expressions for AVP isoforms in humans and the number of tissue infection records for SARS-CoV-2, SARS-CoV, and MERS. For each susceptible tissue, Fig 1 shows the relative mRNA expressions (in PME, Eq 1) of AVPs determined as high (in green) or low (in red) (see Materials and Methods for validation of PME). Furthermore, the regular habitats of each coronavirus were determined based on the highest COD (Eq 2, See Materials and Methods for determination of regularly infected tissues). The lungs and the intestines are regular habitats of SARS-CoV-2 and both tissues contain high PMEs for many APOBEC3 isoforms (Fig 1: A3A, A3B, A3D, A3G, A3H in the lungs, and A3B, A3D, A3G, and A3H in the intestines) and for ZC3HAV1. Similarly, the regular habitats of SARS-CoV (lungs) and MERS (lungs) also contain high PMEs for some APOBEC3 and ZAP isoforms (Fig 1). Therefore, all three surveyed human coronaviruses can regularly infect host tissues where both *ZAP* and *APOBEC3* mRNAs are expressed in abundance and they display no strong preference for tissues deficient in either *ZAP* or *APOBEC3* transcripts.

In an approach similar to Koning et al. [21] and Refsland et al. [22], we acquired the baseline levels of tissue-specific *APOBEC3* from total RNA in many tissues. Fig 1 is consistent with the findings of Koning et al. [21] and Refsland et al. [22], showing that *APOBEC3* mRNAs are abundant in the lung relative to other non-lymphoid tissues. Tissues such as the brain, liver, heart, skeletal muscle, and kidney are all deficient in both *ZAP* and *APOBEC3* mRNAs. The stomach, pancreas and testes abundantly express a subset of APOBEC3 enzymes but are ZAP deficient. In contrast, tissues of lymphoid organs including the spleen, adrenal gland, and thyroid express both AVPs in abundance.

**Fig 2:**
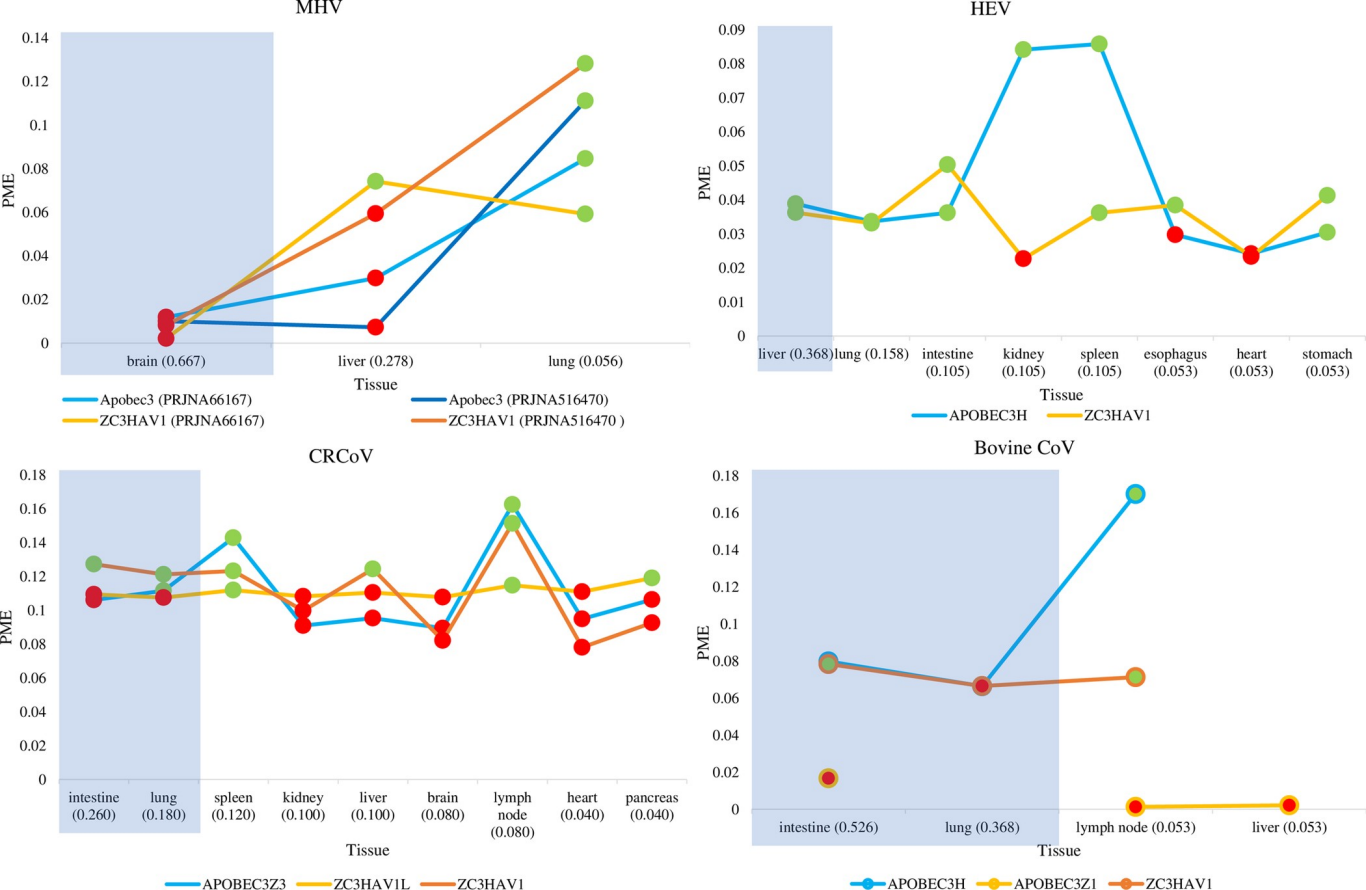
Pig, dog, and cattle tissues that are regularly infected by their respective coronaviruses (HEV, CRCoV, Bovine CoV) have high AVP mRNA expressions, but the mice brain that is regularly infected by MHV does not have high AVP mRNA expressions. The lines show the relative mRNA expressions (PME), for each APOBEC3 and ZAP isoform, among tissues having records of viral infections. Dots highlighted in green and red are PME values that are greater and lower than the averaged PME values, respectively. These PME values were calculated based on averaged mRNA expressions retrieved from the Bovine Genome Database [66], BioProject PRJNA124245 [65], TISSUE 2.0 integrated datasets [64], mouse ENCODE consortium [62] and BioProject PRJNA516470 [63]. For each tissue, the commonness of viral detection (COD) score is appended in brackets next to tissue name. Shaded in light blue-gray are tissues that are regularly infected by the virus (based on highest COD scores).

Retrieving averaged mRNA expression levels of *ZAP* and *APOBEC3* in four other mammalian species (cattle, dog, pig, mice) and their tissue-specific records of coronavirus infection (Figs 2 and S2) reveals the tissues most susceptible to infection for these species (by highest CODs, tissues shaded in blue-gray), as well as the relative mRNA expressions (PMEs) for AVP isoforms in these tissues. Like human coronaviruses, these mammalian coronaviruses also regularly infect tissues exhibiting both high *APOBEC3* and *ZAP* mRNA expressions. Examples include HEV infecting pig liver, CRCoV infecting dog intestines and lungs, and Bovine CoV infecting cattle intestines. Conversely, while MHV regularly infects the brain in mice (Fig 2), PMEs for both *APOBEC3* and *ZAP* are low in this tissue.

Taken together, Figs 1 and 2 show that lungs and intestines are regularly infected by five out of seven surveyed coronaviruses and exhibit high abundances of AVPs in all five mammals, except for lungs in cattle. This suggests that tissue-specific APOBEC3 and ZAP expressions may be correlated. Based on 24 human tissues, PMEs of APOBEC3 and ZAP are significantly positively correlated (e.g., for fitted regression line between 24 A3H and ZC3HAV1 values: coefficient of determination R^2^ = 0.43, P < 0.001). Similarly, we found significant positive correlations between the PMEs of both AVPs in 17 mice tissues (APOBEC3 vs ZC3HAV1: R^2^ = 0.49, P = 0.0017) and ten dog tissues (APOBEC3Z3 vs ZC3HAV1: R^2^ = 0.56, P = 0.021). In contrast, there is no significant correlation between PMEs of both AVPs in 26 cattle tissues (APOBEC3H vs ZC3HAV1: R^2^ = 0.22, P = 0.34) or 33 pig tissues (APOBEC3H vs ZC3HAV1: R^2^ = 0.11, P = 0.065).

### AVPs are expressed in lung epithelial cells, and in particular, the mRNA expressions of *ZAP*, *A3G*, and *ADAR* are upregulated in response to SARS-CoV-2 infection

**Fig 3:**
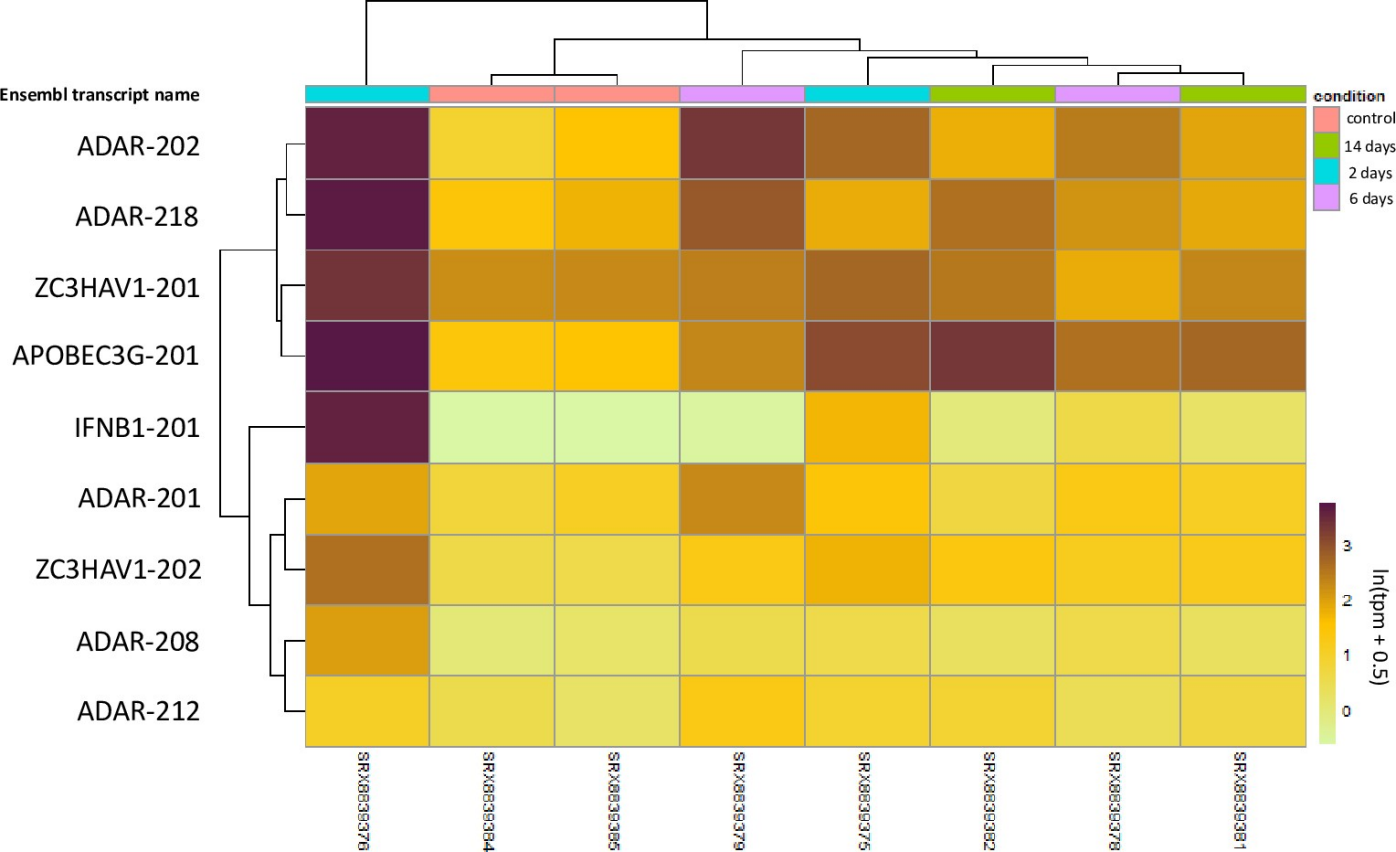
Differential expression of statistically significant transcripts of interest from LoM lung epithelial cell samples at varying time points after infection with SARS-CoV-2. Entries depict ln(tpm + 0.5) transformed fold-changes in kallisto-derived TPM values, from light green (lower) to dark purple (higher). Columns represent each experimental sample with its associated condition (time after infection) shown at the top. Each row represents a particular Ensembl transcript name, which is indicated to the left. Columns and rows are hierarchically clustered by similarity.

Differential transcriptomic analysis of uninfected and infected LoM lung epithelial cell isolates revealed that among ADAR, AID, ZAP, APOBEC1, and APOBEC3 paralogues, transcripts that were found to be significantly differentially expressed between uninfected and infected human lung endothelial cells encoded A3G, ADAR, and ZAP. In all cases, the time after infection (on the time scale considered) was less of a contributing factor to expression levels than the intrinsic presence of infection (Figs 3 and S3). This is evidenced by the consistent clustering of the uninfected control samples contrasted with the greater variance in the transcript-specific clustering of TPMs within infection time points. These results generally support the notion that ADAR, A3G, and ZAP transcripts are either upregulated during SARS-CoV-2 infection relative to uninfected lung epithelial cells or remain at similar levels.

In particular, the results we observed for A3G are consistent with those observed during influenza A infection of A549 lung epithelial cells by Pauli et al. [24] insofar as A3G was upregulated to the exclusion of other APOBEC3 paralogues and a corresponding significant upregulation of IFNβ-encoding transcripts was generally observed in tandem, with the strongest coupling occurring in the samples with the greatest TPM fold-change (Fig 3). Apart from A3G, the sample from the SRX8839376 experiment demonstrated especially high upregulation of all transcripts of interest, followed by SRX8839375 and SRX8839379. All of these are from earlier infection time points (2 and 6 days following infection), suggesting that sharper expression profile changes tend to happen earlier in infection. In contrast, the data make it clear that the cellular response to SARS-CoV-2 infection varies quite substantially. While the control samples have the most closely related expression profiles, the infected samples varied far more widely in their expression profiles and did not cluster strongly in a time-dependent manner. This emphasizes that immunological differences between individuals likely play an important role in combatting infection.

### Coronaviruses targeting tissues with high AVP expressions exhibit decreased CpG and increased U content

**Fig 4:**
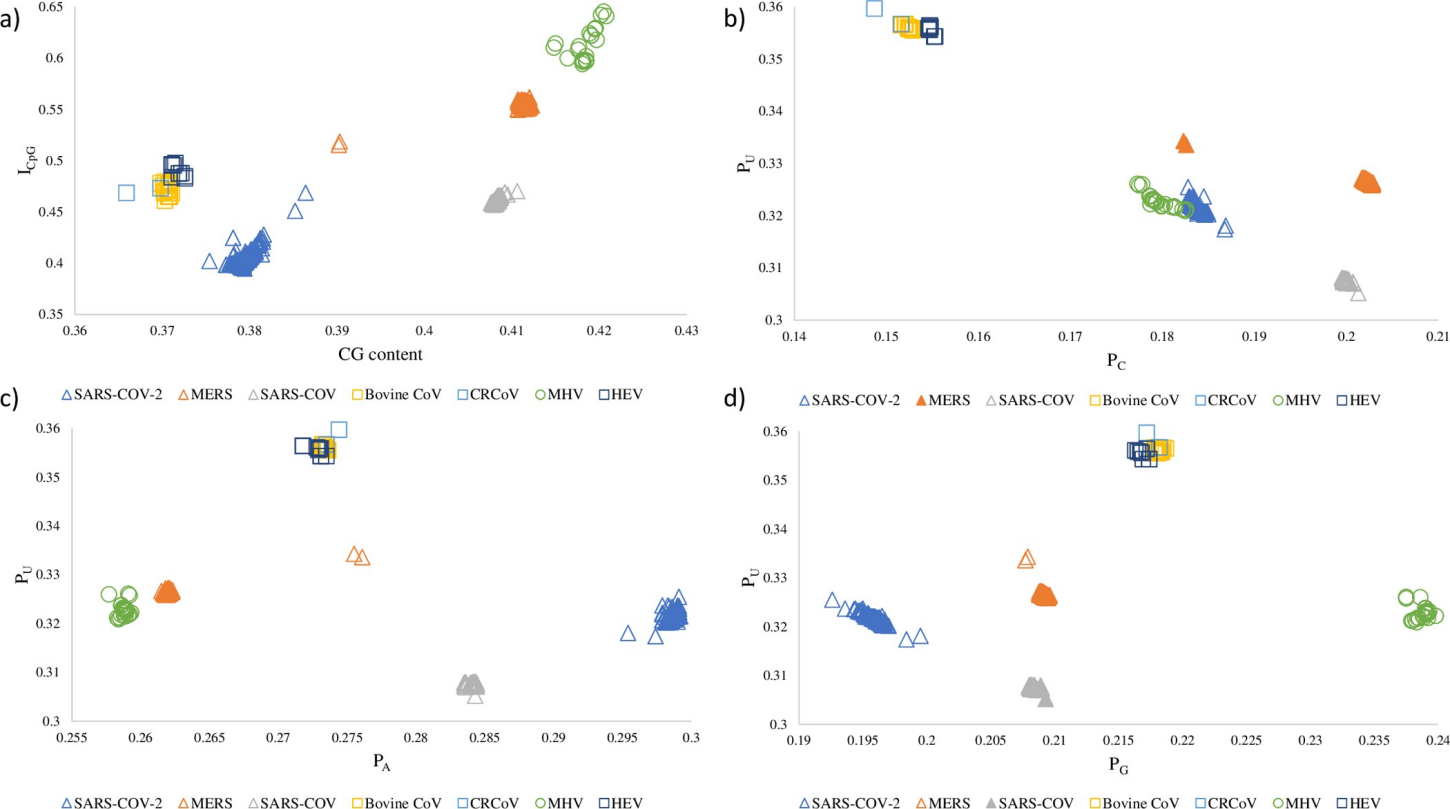
I_CpG_ and nucleotide compositions of seven mammalian-specific coronaviruses. All genomes were MAFFT aligned and all non-conserved sequence ends were trimmed (see Materials and Methods). Panel a) shows that SARS-CoV-2 has the least I_CpG_ in comparison to other coronaviruses from their natural hosts. Panels b), c) and d) compare the proportions of U (P_U_), to those of A (P_A_), C (P_C_), and G (P_G_), respectively. Each panel includes complete and high quality sequence data of 2666 SARS-CoV-2 genomes, 403 MERS genomes, 134 SARS-CoV genomes, 20 Bovine CoV genomes, 2 CRCoV genomes, 26 MHV genomes, and 10 HEV genomes.

Upon comparing the CpG and U contents of coronaviruses, we found those that regularly infected AVP-rich tissues tend to exhibit diminished CpG content in tandem with elevated U content. Conversely, MHV neither targeted AVP-rich tissues, nor did its genome indicate directional mutation with respect to CpG or U content. In both trimmed genomes (Fig 4A) and whole genomes (S4A Fig), MHV had the highest I_CpG_ (about 0.6 or higher) while SARS-CoV-2 had the lowest I_CpG_ (below 0.43 in all but two strains). As for all other coronaviruses surveyed, they also exhibited low I_CpG_ < 0.5 except for MERS being slightly higher. It should be noted that among the seven coronaviruses, I_CpG_ values also showed the greatest variation among MHV genomes but are much more constrained among the other six genomes (S5A Fig). Nonetheless, in all seven coronaviruses, median I_CpG_ is the most deficient among I_XpY_ calculated (where X and Y are A, C, G, or U) and no other dinucleotides display strong deficiency or surplus (S6 Fig).

Fig 4 panels b, c, and d show that the proportion of U nucleotides (P_U_) is inverse to the proportion of C nucleotides (P_C_), but P_U_ does not correlate with P_A_ or P_G_. Bovine CoV, CRCoV, and HEV all have very high P_U_ and conversely very low P_C_. In comparison, MHV does not regularly infect tissues highly expressing APOBEC3 and has relatively reduced P_U_ and increased P_C_ (Fig 4B). Similar to I_CpG_, P_U_ was least constrained in MHV relative to any other coronavirus (S5B Fig). Among human coronaviruses, genomic P_U_ is low in SARS-CoV-2 and MERS and especially in SARS-CoV (Fig 4B). These patterns persisted when I_CpG_ and P_U_ were re-analyzed using whole, untrimmed, genomes (S4B Fig).

### Evidence of C to U directional mutation in SARS-CoV-2 viral regions

**Fig 5:**
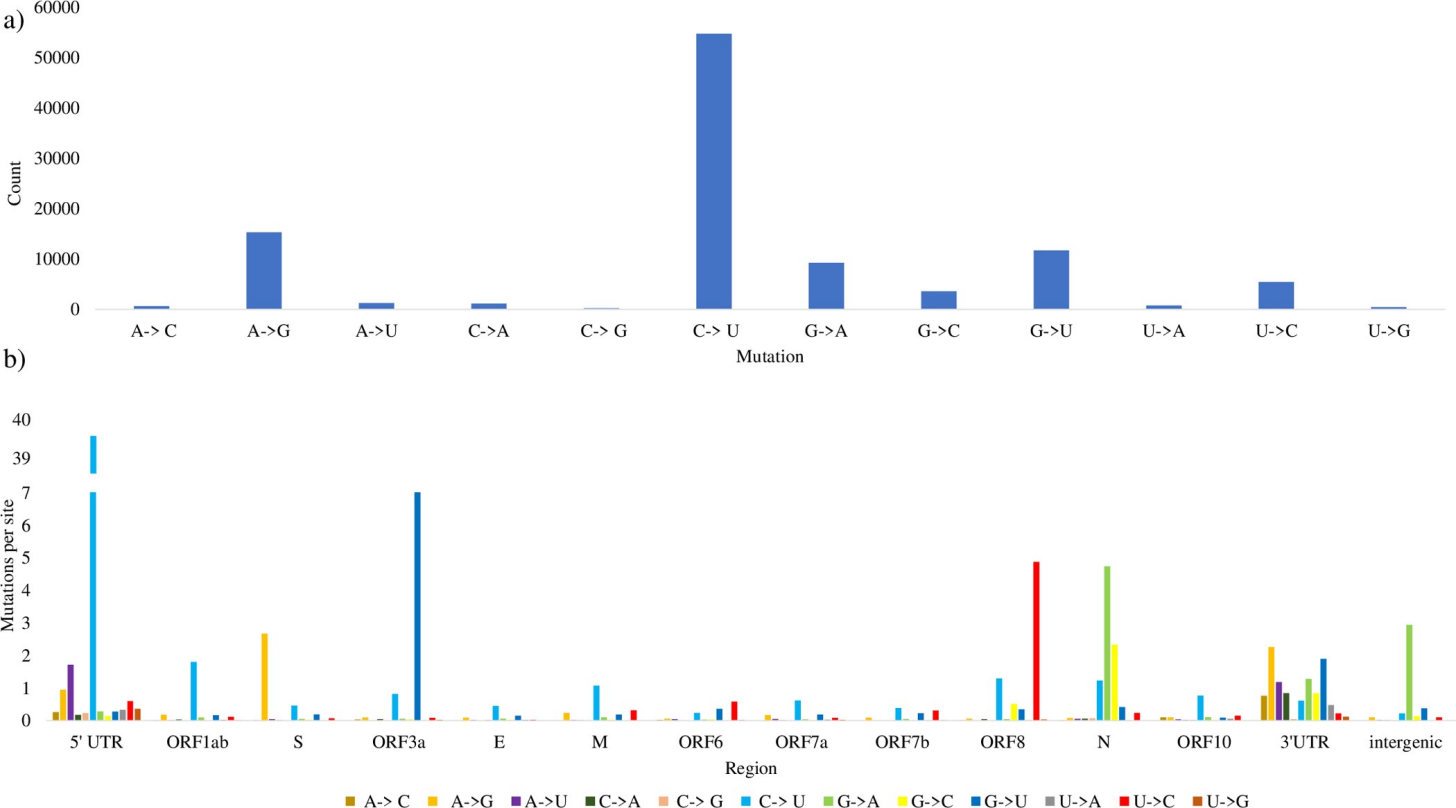
SNPs in 28474 SARS-CoV-2 (complete and incomplete) strains (samples collected up to 5-6-2020), with reference to strain Wuhan-Hu-1 (MN908947, 12-31-2019). Panel a) shows the frequency of each type of mutation in all isolates relative to the reference strain. Panel b) indicates the number of region-specific mutations normalized by region length (Mutations per site = count/sequence length) across all 13 viral regions and at the intergenic spaces. Indels and ambiguous point mutations were omitted from the analysis.

Our above results demonstrate that viral genomes exhibit pronounced shifts towards CpG deficiency and elevated U content when the virus regularly infects tissues with high expression of both AVPs. However, human viruses share similar or lower global P_U_ relative to MHV, which predominantly infects AVP-deficient mice tissues (Fig 4). To better understand the distribution of U content, we examined whether there has been a history of P_U_ elevation in local SARS-CoV-2 regions over the span of the first four months since the virus was first isolated. We observed that most SNPs are C to U mutations (Fig 5A), and these mutations are prevalent at the 5’ UTR and ORF1ab regions but infrequent at other viral regions (Fig 5B).

**Fig 6:**
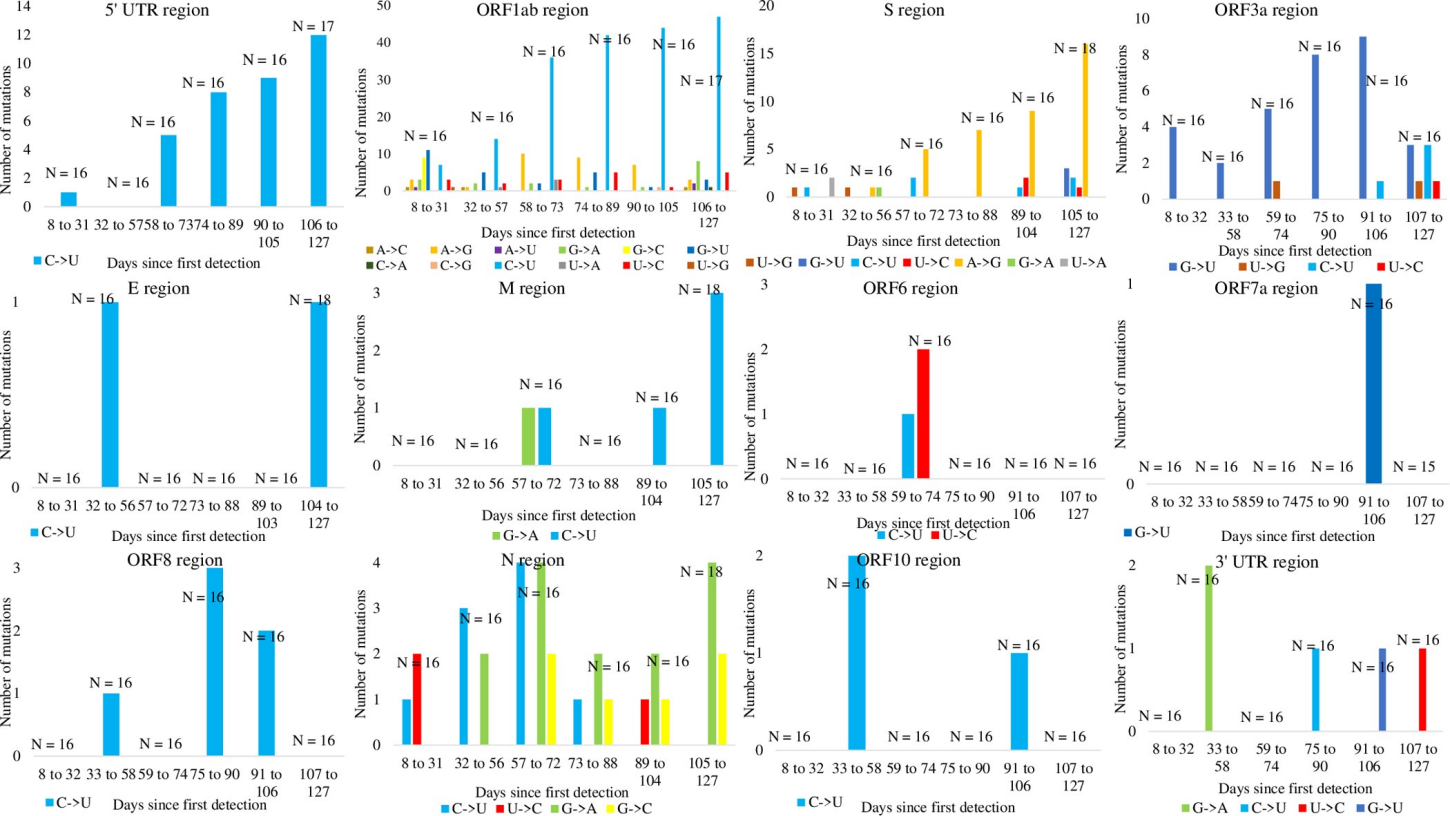
Temporal SNP patterns in 12 SARS-CoV-2 regions. Counts of C to U mutations are most prevalent and increase over time in the 5’ UTR region and ORF1ab region. A to G mutations and G to U mutations are favoured in the S and ORF3a regions, respectively. In the eight other SARS-CoV-2 viral regions, SNPs are infrequent and display no obvious preference. A total of 99 complete and high-quality SARS-CoV-2 genomes with complete NCBI annotations were selected. These genomes were picked because they were each collected on a unique date, from the earliest sequenced strain Wuhan-Hu-1 (MN908947, 12-31-2019) to strain mink/NED/NB04 (MT457401, 5-6-2020), and each strain was grouped into one of six time ranges with equal sample size. N denotes the number of strains per time range.

We next assessed temporal SNP patterns in each of 12 SARS-CoV-2 regions (excluding ORF7b) from a sample of 99 SARS-CoV-2 strains (see Materials and Methods). We observed a striking number of C to U mutations in aligned sequences between the reference and sampled strains (Fig 6), and the total number of C to U mutations trends upward over time in the 5’ UTR and ORF1ab regions, but other regions did not exhibit any clear C to U mutation patterns. Other notable substitution patterns were observed in the S region and ORF3a regions, namely: A to G mutations and G to U mutations, respectively.

**Fig 7:**
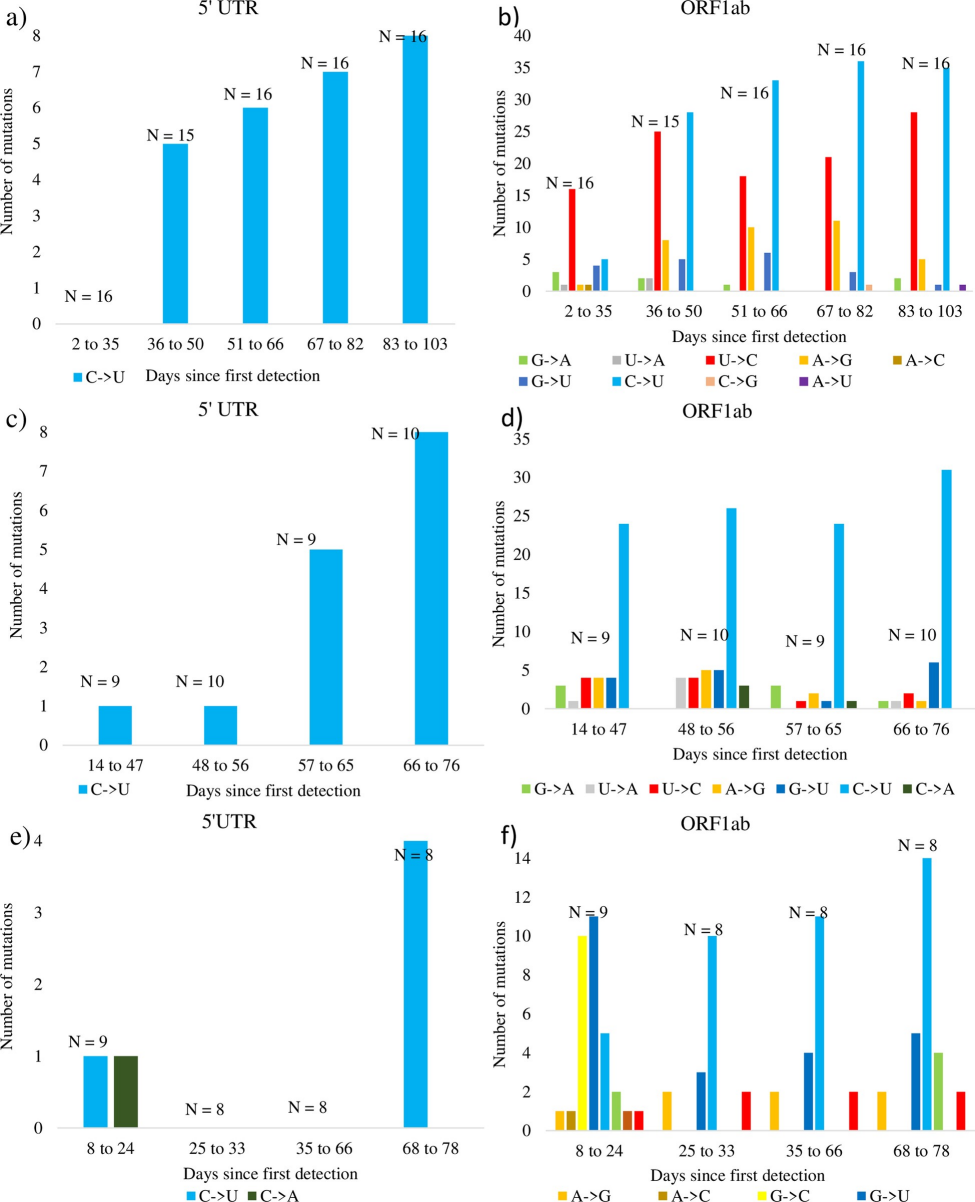
Geographical and temporal SNP patterns for samples of complete and high-quality SARS-CoV-2 strains. Panels respectively show SNPs within the 5’ UTR and ORF1ab regions relative to the first isolate collected within the country. Panels a) and b) show SNPs from 79 strains relative to accession MN985325 collected in the United States. Panels c) and d) show SNPs from 38 strains relative to accession MT450920 collected in Australia. Panels e) and f) show SNPs from 33 strains relative to accession MN908947 collected in China.

To control for potential geographical bias, such as a widespread C to U hypermutation before SARS-CoV-2 was transmitted outside of China, we show that the prevalence of C to U mutations in 5’ UTR and ORF1ab regions persists when considering the temporal and geographical SNP patterns of SARS-CoV-2 strains with unique sample dates are isolated from three different countries: United States, Australia, and China (Figs 7 and S7–S9). In all three countries, aligned sequences revealed the same pattern of C to U mutations we previously observed. Likewise, C to U mutations trended upwards over time in the 5’ UTR and ORF1ab (Fig 7), but this bias was absent in other regions (S7–S9 Figs). The number of C to U mutations is significantly greater than for any other mutations in the ORF1ab region regardless of geographical constraint (all ORF1ab panels in Figs 5 and 6, Wilcoxon rank sum test with continuity correction: P < 0.01). Indeed, only C to U mutations were observed in the 5’ UTR region of strains collected from the United States and Australia (Fig 7), but no two countries shared the same SNP patterns in other viral regions (S7–S9 Figs).

We next investigated the sequence context for the prevalently observed C to U mutations. A total of 477 SNPs were observed comparing the reference genome (Wuhan-Hu-1) to a sample of 98 SARS-CoV-2 strains. Over half of these SNPs were C to U mutations (262/477, S1 Table), and 144 and 82 C to U mutations were synonymous and non-synonymous substitutions, respectively. These 262 C to U mutations were found at 98 unique nucleotide sites with respect to the reference genome of Wuhan-Hu-1 (S10 Fig), with 92 unique sites located within protein coding regions, 55 of which accounted for the synonymous substitutions and 37 sites were associated with non-synonymous substitutions. The remaining six unique mutation sites were not located within protein coding regions. Furthermore, the locations of unique sites subjected to C to U mutations were roughly evenly distributed across the Wuhan-Hu-1 genome (S10A Fig); these SNPs were not densely packed at any specific sequence region (S10B Fig).

### C to U mutations in SARS-CoV-2 occur at known APOBEC3 recognition sites

Many of the aforementioned 98 unique C to U mutations sites occur at 5’ CC and 5’ UC (with C to U mutation sites underlined) dinucleotides embedded in motifs that facilitate APOBEC3 binding and editing in HIV-1, MLV, and SIV (Table 2). For each dinucleotide and trinucleotide motif, odds-ratios were calculated using the observed proportion of C to U mutations divided by the expected proportion of C to U mutations (see Materials and Methods). Among dinucleotides, only 5’CC and 5’ UC have odds-ratios > 1 (observed > expected) with 1.136 and 1.111, respectively. In viruses such as HIV, MLV, and SIV, most studies are consistent in demonstrating that A3G prefers to edit 5’ CC whereas the other six APOBEC3 enzymes prefer to edit 5’ TC (Table 2).

**Table 2:** The preferred motif contexts of C to U mutations in the SARS-CoV-2 genome are the same as those previously identified in HIV, MLV, and SIV that were subjected to editing by APOBEC3 enzymes.

Motifs	Hotspots subjected to editing in HIV, MLV, and SIV*	Motifs with C to U mutations in Wuhan-Hu-1	Total motifs in Wuhan-Hu-1	Odds-ratio
AC	A3D^5^	36	2023	0.997
AAC		9	615	0.82
CAC		9	459	1.099
GAC		3	340	0.494
UAC	AID^7^	15	609	1.38
CC	A3G^4^^,^^8^	18	888	**1.136**
ACC		11	376	**1.639**
CCC	A3G^1^^,^^2^^,^^3^^,^^6^^,^^7^^,^^14^	3	116	**1.449**
GCC		0	187	0
UCC	A3A^11^, A3G^2^	4	209	1.073
GC		16	1168	0.768
AGC		5	301	0.931
CGC		2	97	1.155
GGC		2	223	0.503
UGC		7	547	0.717
UC	A3A^9^, A3B^6^^,^^12^, A3D^5^^,^^8^, A3F^2^^,^^6^^,^^7^^,^^8^^,^ ^15^, A3H^8^^,^^13^^,^^16^, A1^17^	28	1413	**1.111**
AUC		3	339	0.496
CUC		4	287	0.781
GUC		5	269	1.042
UUC	A3C^10^^,^^14^, A3F^3^^,^^14^, A3H^18^	16	518	**1.731**

Specifically, the preferred dinucleotides are 5’ CC and 5’ UC, and the preferred trinucleotides are 5’ ACC, 5’ CCC, and 5’ UUC (by highest odds-ratio > 1 in bold). Underlined are sites subjected to C to U mutations. In red are non-APOBEC3 deaminases that were reported to have C to U/T editing ability in non-viral sequences.

^1^ [79];

^2^ [51];

^3^ [80];

^4^ [42];

^5^ [44];

^6^ [41];

^7^ [81];

^8^ [40];

^9^ [82];

^10^ [83];

^11^ [84];

^12^ [85];

^13^ [86];

^14^ [43];

^15^ [39];

^16^ [87];

^17^ [88];

^18^ [89]

* All preferred motifs subjected to RNA editing by APOBEC3 are based on HIV-1 mutagenesis studies except for ^4^ [42] which studied MLV. ^2^ [51], ^6^ [41], and ^14^ [43] additionally studied MLV and ^5^ [44] additionally studied SIV. Consensus motif for AID enzyme editing was determined from a mutagenesis study of *rpoB* gene in *Escherichia coli*: ^7^ [81], and consensus motif for APOBEC1 enzyme editing was determined from mutagenesis study of chicken B-cell line DT40: ^17^ [88].

In the context of 5’ NCC, the two most preferred trinucleotide motifs are 5’ ACC (odds-ratio = 1.639) and 5’ CCC (odds-ratio = 1.449). This observation is consistent with multiple studies showing that 5’ CCC is preferred [41, 79–81], although others found that 5’ RCC may also be preferred [51] by A3G editing in HIV-1 and MLV. When 5’ NUC is considered, the preferred trinucleotide in SARS-CoV-2 is 5’ UUC (odds-ratio = 1.731). This observation is also corroborated by multiple studies indicating that 5’ TTC is preferred by all APOBEC3 enzymes except A3G [45–47].

We further considered C to U mutations in the context of 5’ NCCN and 5’ NTCN. All studies summarized in Table 3 conclude that both the -2 and +1 positions flanking 5’ NC influence the efficacy of APOBEC3 editing. Comparing between reported APOBEC3 enzyme activities by independent studies, activity levels were classified as preferred (++), less preferred (+), inefficient (-), and avoided (—) among motifs examined. A3D was excluded from Table 3 because its consensus target could not be specified beyond 5’ (T/A)(T/A)C(G/T) [7]; an A3D-preferred motif has not been established [52] because the catalytic properties of this enzyme are not fully characterized [90]. Despite the lack of a strongly preferred consensus sequence among many APOBEC3 enzymes, most studies are consistent in reporting 5’ CCC(A/T) as preferred targets of A3G, and 5’ TTC(A/T) are among, if not the most, preferred motifs by all 5’ TC editing APOBEC3 enzymes except A3B (Table 3).

**Table 3:** Number of unique C sites, in the context of 5’ N(U/C)CN motifs, that were subjected to mutation when the genome of Wuhan-Hu-1 was compared to 98 later sampled SARS-CoV-2 strains.

**Motifs**	**Edited motifs**	**Motifs in genome**	**Odds-ratio**	**Consensus motif reports**^**a**^	**A3A**^**b**^ **[55] /[52]**	**A3B [52]**	**A3C [52]**	**A3F [91]/[53]/[52]**	**A3H [52]/[89]**	**UC (stem)**	**UC (loop)**	**UC (open)**	**A3 preferred structure**
AUCA	1	137	0.368		--/++	-	-	+/-/-	+/na		1		open [52]: A, B, C, D, G, H
AUCC	1	51	0.987		na/na	na	na	-/na/na	na/na			1	
AUCG	0	20	0.000		--/na	na	na	-/na/na	na/na				loop [56]: A
AUCU	1	130	0.387		-/na	na	na	-/na/na	na/na		1		
CUCA	3	131	1.153	B [41], C [41], F [41]	na/++	-	--	-/--/++	++/na			3	
CUCC	0	30	0.000		na/-	--	-	-/na/-	-/na				open [52]: A, B, C, F, H
CUCG	0	32	0.000		na/-	-	-	--/na/--	-/na				
CUCU	1	93	0.541		na/+	+	+	-/na/--	++/na			1	
GUCA	3	98	1.542	B [41], C [41]	+/+	++	+	--/-/-	-/na		1	1	
GUCC	1	47	1.071		na/na	na	na	na/na/na	na/na	1			
GUCG	0	21	0.000		-/--	--	--	na/na/+	--/na				loop [52]: A, B, G; loop [56]: A; open [52]: F, H
GUCU	1	103	0.489		--/na	na	na	--/na/na	na/na	1			
UUCA	7	182	1.937	F [51]	++/na	na	na	++/++/na	na/++		1	6	
UUCC	0	80	0.000		na/na	na	na	+/+/na	na/na				
UUCG	3	39	3.874		+/na	na	na	-/++/na	na/-	1	2		loop [56]: A
UUCU	6	216	1.399	F [51,80]	+/++	--	++	+/-/++	+/++			5	open [52]: A, B, C, F, H
**Motifs**	**Edited motifs**	**Motifs in genome**	**Odds-ratio**	**Consensus motif reports**	**A3G [79]/[39]/[91]**					**CC (stem)**	**CC (loop)**	**CC (open)**	**A3G preferred structure**
ACCA	4	151	1.302		-/na/+							3	
ACCC	1	55	0.894		na/na/-					1			
ACCG	1	29	1.695		-/na/+							1	
ACCU	5	139	1.769		-/na/-					1	2	1	
CCCA	0	39	0.000	G [41,42,51,80]	++/++/++								
CCCC	1	14	3.512	G [51]	na/-/+							1	
CCCG	0	13	0.000		++/na/+								loop [56]: G
CCCU	2	49	2.007	G [51]	+/na/+						1		
GCCA	0	78	0.000		--/--/--								
GCCC	0	17	0.000		na/na/--								
GCCG	0	17	0.000		na/na/na								
GCCU	0	75	0.000		na/na/na								
UCCA	2	86	1.143	G [51]	na/na/+					1		1	
UCCC	0	29	0.000	G [51]	na/na/-								
UCCG	0	15	0.000		na/na/-								
UCCU	2	79	1.245	G [42,51]	na/na/-						1		

Underlined are the mutated C sites. “Motifs in genome” indicates the number of observed motifs in the genome of Wuhan-Hu-1. Column 5 shows the consensus 5’ N(U/C)CN motifs as reported by surveyed studies. Columns 6 to 10 show the relative levels of APOBEC3 editing activities at select 5’ N(U/C)CN motifs tested by studies. Columns 11 to 13 show the structural configurations of 5’ (U/C)C dinucleotide sites in Wuhan-Hu-1 where mutations had occurred in later strains. The last column shows the preferred structural configurations of 5’ (U/C)C edited by APOBEC3 as reported by surveyed studies.

^a^–The seven APOBEC3 members were abbreviated in the table to show only the last letter of the enzyme (e.g., A3A = A).

^b^–The relative APOBEC3 activity levels at surveyed motifs were designated with + and–symbols: “++” = preferred, “+” = less preferred, “–” = inefficient, “––” = avoided. The “na” indicates that data is unavailable. Activity levels reported by different studies are separated by a “/” symbol, and the order of activity data corresponds to the order of cited references shown in the table header.

In SARS-CoV-2, the two tetranucleotides embedding the 5’ UC editing target with the highest number of unique C to U mutations were 5’ UUCA and 5’ UUCU (odds-ratios 1.937 and 1.399, respectively), followed by 5’ GUCA, 5’ CUCA, and 5’UUCG (odds-ratios 1.542, 1.153, 3.874, respectively), and all except 5’UUCG are preferred APOBEC3 editing motifs (Table 3). However, the A3G-preferred 5’ CC motifs 5’ CCC(A/U) (e,g. [91]) were not found and 5’ ACC(A/U) were instead abundant in SARS-CoV-2 (Table 3). Nevertheless, 5’ GCCN were avoided and no such motifs were observed in SARS-CoV-2.

Additionally, McDaniel et al. [52] showed that the 5’ UC targets in 5’ NUC(A/U) motifs highly preferred an open structure configuration. Similarly, in SARS-CoV-2, the 5’ UC within 5’ NUC(A/U) motifs highly preferred open structure configurations (Table 3: 16 out of 23, see secondary structure details in S6 File). Of 5’ (C/U)C targets in 5’ N(C/U)CG that were reported to prefer the loop region by Sharma and Baysal [56], three 5’ NUCG motifs were observed in SARS-CoV-2 (all being 5’ UUCG), two were found in the loop region and one was found in the stem region; in contrast, only one 5’ NCCG (5’ ACCG) motif was observed and it had an open structure (Table 3).

### CpG deficiency is maintained in specific viral regions and I_CpG_ does not differ notably among SARS-CoV-2 genomes collected in the span of four months

**Fig 8:**
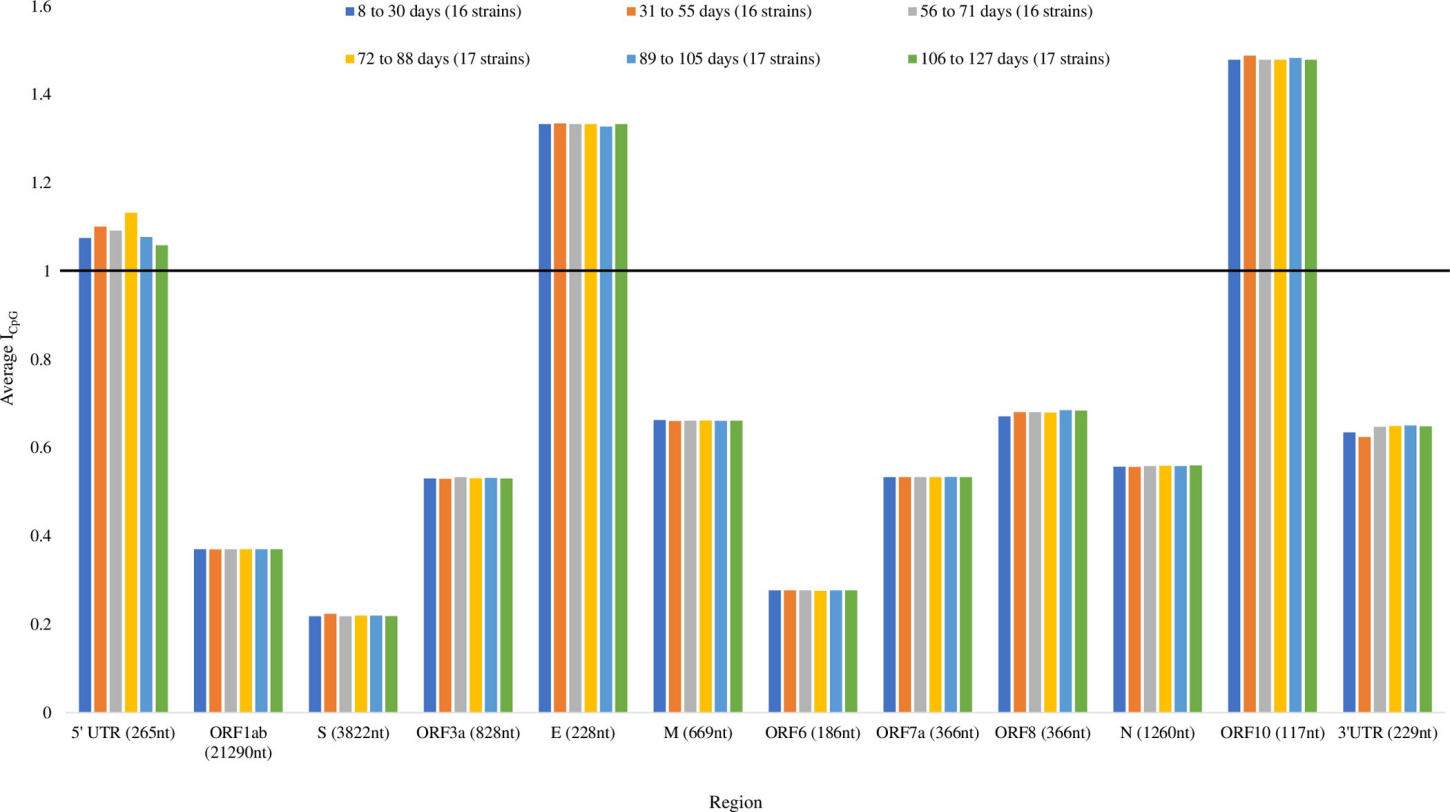
A temporal analysis for local I_CpG_ in a sample of 99 complete and high-quality SARS-CoV-2 strains with complete NCBI annotations. Each retrieved strain was collected on a unique day, regardless of geographical location, since the first isolated strain Wuhan-Hu-1 (MN908947, 12-31-2019) to strain mink/NED/NB04 (MT457401, 5-6-2020). Each strain was grouped into one of six time ranges with approximately equal sample size. I_CpG_ did not change substantially over the 127 days since first detection, but I_CpG_ values were not uniform across viral regions. I_CpG_ values were lowest in ORF1ab, S, and ORF6 regions, and the highest in the 5’ UTR, E, and ORF10 regions. The horizontal black line highlights I_CpG_ = 1.

Lastly, we performed a temporal analysis to determine whether there are differences in I_CpG_ within and between viral regions among the 99 SARS-CoV-2 strains. Within viral regions, there were no notable differences in I_CpG_ between strains sampled at different time intervals (Fig 8). However, there were notable differences in I_CpG_ between viral regions. In particular, the ORF1ab, S, and ORF6 regions had the lowest I_CpG_ values < 1, whereas the 5’ UTR, E, and ORF10 regions had the highest I_CpG_ values > 1. Thus, CpG content varies substantially across the SARS-CoV-2 genome [92, 93].

Next, we examined the CpG content in the SARS-CoV-2 genome in the context of CN_X_GNCG motifs that were preferably recognized by ZAP in mice [16]. When the reference Wuhan-Hu-1 was compared to the other 98 strains sequenced in the following four months, there were only 11 unique sites where either C or G, in the context of CpG, had been mutated, and only two out of the 11 mutations occurred in the context of CN_X_GNCG. In addition, other C or G mutations, in the context of CpG, do not particularly prefer CG-rich sequences (S6 File). Note that we determined the number of mutations that have occurred at unique sites when referenced to the genome of Wuhan-Hu-1, because while a mutation at a given site may be carried by multiple later strains, the creation of such mutations could be derived from singular events. Nevertheless, there was a deficit in the total number of observed CG dinucleotides (Table 4: Obs/Exp ratio = 0.408) and CN_X_GNCG motifs (Table 4: Obs/Exp ratio ranges from 0.309 to 0.619) at the genome of Wuhan-Hu-1.

**Table 4:** Total number of unique C and G mutations and those that occurred in the context of CpG motifs in SARS-CoV-2.

Nucleotides and Motifs	Number of unique mutations at C or G	Observed number	Expected number	Obs/Exp ratio
**C**	98	5492		
**G**	66	5863		
**CG**	11	439	1076.802	0.408
**GNCG**	3	85	211.104	0.403
**CN**_**4**_**GNCG**	0	12	38.777	0.309
**CN**_**5**_**GNCG**	0	24	38.780	0.619
**CN**_**6**_**GNCG**	1	14	38.783	0.361
**CN**_**7**_**GNCG**	1	17	38.785	0.438
**CN**_**8**_**GNCG**	0	22	38.788	0.567

Number of unique mutations were determined on the Wuhan-Hu-1 genome when it is compared to 98 later strains, in the context of CN_X_GNCG (underlined are either C or G nucleotides that were mutated). N_X_ indicates the spacer sequence of length x = 4 nt to 8 nt. The “Observed number” indicates the total number of nucleotide and motifs observed in the genome of Wuhan-Hu-1, the “Expected number” is calculated based on the total nucleotide frequencies and the length of Wuhan-Hu-1 genome, and “Obs/Exp ratio” calculates Observed number/Expected number. The specific sequence contexts of all 11 unique C or G mutations that occurred at CpG are shown in S6 File.

## Discussion

SARS-CoV-2 poses a serious global health emergency. Since its outset in Wuhan City, Hubei province of China in December 2019, the viral outbreak has resulted in over 20 million confirmed cases around the world (https://www.who.int/emergencies/diseases/novel-coronavirus-2019, last accessed August 12, 2020). The pandemic has prompted an immediate global effort to sequence the genome of SARS-CoV-2, and by May 2020 over 28000 strains have been publicly deposited over the course of just four months. With a wealth of sequence data, we performed a comprehensive comparative genome study on SARS-CoV-2 and six other coronaviruses across five mammalian species, with the aim to understand how coronaviruses evolve in response to tissue-specific host immune systems.

We tested whether APOBEC3 and ZAP immune responses act as selective pressures to shape the genome of an infecting coronavirus. We note that ZAP is highly expressed in human lungs (Fig 1) and we observed that its expression is further upregulated in SARS-CoV-2 infected lung epithelial cells relative to the control (Fig 3). Our observations are compatible with the notion that cytoplasmic ZAP can bind to CpG dinucleotides to facilitate the degradation of viral transcripts. This idea, in conjunction with our observations, is corroborated by a recent study that found ZAP targets CpG to restrict SARS-CoV-2 replication in human lung cells [17]. In contrast to ZAP, APOBEC3 enzymes are mostly expressed in immune cells such as CD4^+^ T cells residing in tissues [22]. SARS-CoV-2 infection triggers T cell response in infected patients [94], and the ability of CD4^+^ T cells to recognize a virus would then allow APOBEC3 enzymes to be packaged into the virions and cause RNA-editing [20].

We predicted that viral genomes should be driven towards reduced CpG dinucleotides to elude ZAP-mediated cellular antiviral defense, and increased U residues because of RNA editing by APOBEC3 proteins. In line with our expectations, we found compelling hallmarks of CpG deficiency as well as elevated U with lowered C contents in the genomes of SARS-CoV-2, SARS-COV, MERS, Bovine CoV, CRCoV, and HEV that regularly infected mammalian tissues expressing both AVPs in abundance (Fig 4). Unsurprisingly, these sequence trends were absent from MHV genomes (Fig 4) as this virus regularly infects mice tissues that lowly express AVPs (Fig 2). Corroborating this observation, both I_CpG_ and P_U_ values showed the greatest variation among MHV strains (S5 Fig), suggesting that MHV genomes are not constrained by either AVP. These results suggest that when a virus regularly infects host tissues that are abundant in ZAP and APOBEC3, these AVPs shape the molecular evolution of viral genomes in two ways: CpG deficiency allows the virus to evade ZAP-mediated antiviral defense, and elevated U content due to APOBEC3 editing activity.

Among three human coronaviruses, SARS-CoV-2 genomes exhibit the most CpG deficiency (Fig 4A). Many recent studies point to Bat CoV RaTG13 as the most closely related known relative of SARS-CoV-2 when the whole genome is considered [95–98], and to the bat *Rhinolophus affinis* as a potential intermediate host or reservoir for SARS-CoV-2 [99]. Indeed, the I_CpG_ values in SARS-CoV-2 are comparable to that of Bat CoV RaTG13 infecting *Rhinolophus affinis* but lower than that of many other coronaviruses surveyed [18].

Nevertheless, global CpG is not more deficient in SARS-CoV-2 than many other highly pathogenic coronaviruses [92]. Despite this, CpG deficiency largely fluctuates in local coding regions [92, 93, 100]; the S, ORF1ab, and ORF6 regions have the most severe CpG deficiencies (Fig 8, I_CpG_ < 0.4), whereas the 5’ UTR, E, and ORF10 regions have CpG surplus with no signs of CpG deficiency (Fig 8, I_CpG_ > 1). This may be surprising since one would expect that maintaining high CpG, regardless of its location, should have a detrimental effect on the virus. However, ZAP-mediated RNA degradation is cumulative [7]. When CpG dinucleotides are added to individual viral segment 1 or 2 in HIV-1, the inhibitory effect of ZAP is weak, but when the same CpG dinucleotides are added to both segments 1 and 2, the ZAP inhibition effect is strong. This implies that longer RNA sequences (ORF1ab and S) are more likely to be targeted by ZAP.

Moreover, a study on early SARS-CoV-2 genome evolution [92] suggests that the CG-rich N region is biased towards mutations lowering CpG content, whereas the CpG levels remain consistently low in the S region. Nonetheless, we found no notable change in I_CpG_ between 99 SARS-CoV-2 strains sampled in four months since its first detection (Fig 8), and occurrences of unique mutations at the CG dinucleotides in the context of the CG-rich CN_X_GNCG motifs known to be preferred for ZAP targeting in mice [16] were rare (Table 4). This suggests that the evolutionary adaptation to CpG deficiency had not been a rapid process. Despite this, the first isolated SARS-CoV-2 genome is deficient in both CG dinucleotides and CN_X_GNCG motifs (Table 4). Hence, SARS-CoV-2 may have preadapted to a low-CpG human environment as its closest RaTG13 counterpart is likewise CpG deficient [17]. Altogether, our results are consistent with recent studies suggesting that, similar to the HIV-1 genome [7], the SARS-CoV-2 genome appears to be CpG deficient to evade ZAP recognition [17].

While APOBEC3 enzymes are highly expressed in immune cells [21, 22], they are also detected in mammary and lung epithelial cells [23, 24]. An analysis of total RNA at the tissue level (Fig 1) showed that APOBEC3 enzymes are highly expressed in healthy lung tissues in comparison to other non-lymphoid tissues; this is consistent with results reported by Koning et al. [21] and Refsland et al. [22]. Indeed, expressions of APOBEC3 enzymes are not confined to immune organs but are dependent on tissue lymphocyte contents. To further localize antiviral protein expression, we examined the transcriptomic data of human lung epithelial cells in the presence and absence of SARS-CoV-2 infection (Fig 3). Our findings were consistent with Pauli et al. [24] insofar as we observed selective upregulation of A3G to the exclusion of other APOBEC3 paralogues in the lung epithelial cells during viral infection. This suggests that tissue-residing immune cells are predominantly contributing to the APOBEC3 levels observed in tissue total RNA (Fig 1), particularly with respect to the high abundance of A3A in lung tissue. It remains likely that tissue-residing immune cells are primarily responsible for variable APOBEC3 expressions at the tissue level.

A survey of complete SARS-CoV-2 genomes did not indicate drastically increased U and decreased C contents (Fig 4B). Nonetheless, over the span of four months since the virus was first isolated, there has been a history of P_U_ elevation and strong bias for C to U mutations relative to other substitutions. These C to U mutations are mostly located in the 5’ UTR and ORF1ab regions (Figs 5–7), accounting for over half of all SNPs in SARS-CoV-2. That we observed the same prevalence of C to U mutations in the 5’ UTR and ORF1ab regions in strains collected from three different countries (Fig 7) suggests that geographic patterns of sampling were not a confounding factor. Indeed, these results suggest that SARS-CoV-2 is consistently biased towards C to U mutations.

Consensus motifs embedding C to U mutations that are acted on by APOBEC3 enzymes have been experimentally verified in HIV-1. To support the hypothesis that C to U mutations in SARS-CoV-2 are catalyzed by APOBEC3 enzymes, we determined that the preferred C to U mutation hotspots in SARS-CoV-2 are the same as those in HIV-1. As summarized in Table 2, most studies have shown that 5’ CC and 5’ CCC (with C to U mutation sites underlined) are the preferred consensus motifs subjected to RNA editing by A3G in HIV-1 and MLV. As for other APOBEC3 enzymes, 5’ TC and 5’ TTC are the preferred consensus motifs that are subjected to RNA editing in HIV-1, MLV, and SIV. Similarly, C to U mutations are prevalent in the aforementioned sequence contexts in SARS-CoV-2, suggesting that APOBEC3 enzymes may indeed edit the SARS-CoV-2 genome. Furthermore, among 5’ N(C/U)CN mutations in SARS-CoV-2, the APOBEC3-preferred 5’ UUC(A/U) were the most commonly observed (Table 3) and the embedded 5’ UC targets preferred an open structure, akin to what was shown by McDaniel et al. [52]. However, the two 5’ CCC(A/U) motifs that are preferably edited by A3G (Table 3) were not found and 5’ NCCG motifs did not prefer the loop region as shown by Sharma and Baysal [56]. As APOBEC3 enzymes can be efficiently co-packaged into the same viral particle [50], these results suggest that while all A3A, A3B, A3C, A3F, and A3H could contribute to the prevalence of 5’ UC to 5’ UU mutations, the effect of A3G weakly explains 5’ CC to 5’ CU mutations in SARS-CoV-2. This is consistent with the observation made by Pauli et al. [24] regarding A3G exhibiting no antiviral efficacy during influenza A infection despite its upregulation in that context.

It is not excluded that other deaminase enzymes may contribute to RNA editing in SARS-CoV-2. The preferred consensus motif that is subjected to editing by AID is 5’ UAC in the *rpoB* gene in *E*. *coli* [81]; this may explain why C to U mutations were also preferred at 5’ UAC in SARS-CoV-2 (Table 2). In addition, 5’ TC is preferentially edited by APOBEC1 in chicken B-cells [88]. Nonetheless, it is unknown whether these enzymes possess the ability to target viral genomes [81, 88]. Another noteworthy observation is that A to G mutation was preferred in the S region and the numbers of A to G mutations in this specific region were increasing over time (Fig 6). This mutation may be caused by the ADAR enzyme [34, 38], which edits A into I and subsequently into G, in viruses that infect the lungs such as Influenza virus A and Measles virus [101, 102]. Although ADAR was known for targeting double-stranded RNAs and not single-stranded RNAs [35, 103–105], the secondary structure of viral genomes often contains local regions of base-pairings as possible substrate for ADAR. A survey of APOBEC1, AID, and ADAR expressions in 27 human tissues (S11 Fig) shows that APOBEC1 and AID, but not ADAR, are most expressed in the small intestines. None are highly expressed in the lungs, but *ADAR* mRNA expression was upregulated in SARS-CoV-2 infected lung epithelial cells in comparison to the control (Fig 3 and S3 Fig). Therefore, in addition to APOBEC3, other host deaminase systems such as A1, AID, and ADAR may act to edit the genome of SARS-CoV-2; however, both AID and A1 deaminases are DNA mutators that are not known to target viruses [81, 88].

The current study focuses on the evolutionary pressure that host immune systems exert onto viral genomes. Our aim is to prompt motivations for vaccine designs in the development of attenuated RNA viruses. Previous experimental works have shown that increasing CpG dinucleotides in CpG-deficient viral genomes drastically decrease viral replication and virulence [10, 106–110], and in recent years several studies have proposed vaccine development strategies involving increased CpG to attenuate RNA viruses [5, 10, 107, 109]. Increasing CpG content may provide a good starting point for strategies to attenuate SARS-CoV-2. On the other hand, because C to U deamination cannot be proof-read by viral exonuclease Nsp14-ExoN [36, 111, 112], host innate deaminases may drive up the rate of evolution in viral genomes [34, 49]. The possibility of APOBEC3 editing activity and its potential influence on the pathogenesis and drug resistance of viruses such as SARS-CoV-2 in the long term requires further investigation and scrutiny.

## Supporting information

S1 Fig Averaged human tissue-specific mRNA expressions (in FPKM and TPM) from four independent studies (PRJEB4337, PRJEB2445, PRJNA280600, and GTEx) and regular tissue habitats of SARS-CoV, SARS-CoV-2 and MERS (in the last three columns). The color spectrum from blue (higher) to white (median) to red (lower) indicates the comparative tissue-specific mRNA expressions of seven APOBEC3 isoforms (A3A, A3B, A3C, and A3D, A3F, A3G, A3H) and two ZAP isoforms (ZC3HAV1 and ZC3HAV1L) within each independent study. Similarly, for tissue habitats of the viruses, the color spectrum (from more blue to white) indicates the prevalence of tissue infection observed from independent studies (observed most commonly to least commonly across different studies, respectively). Light grey indicates tissues with no expression data or for which we encountered no peer-reviewed reports of infection.
(TIF)

S2 Fig Averaged host tissue-specific mRNA expressions (in FPKM and TPM) and regular tissue habitats of Murine MHV (MHV), Bovine CoV, Canine CoV (CRCoV), and Porcine HEV (HEV). The color spectrum from blue (higher) to white (median) to red (lower) indicates the comparative tissue-specific mRNA expressions of APOBEC3 and ZAP (ZC3HAV1) isoforms within each independent study. Similarly, for tissue habitats of the viruses, the color spectrum (from more blue to white) indicates the prevalence of tissue infection observed from independent studies (observed most commonly to least commonly across different studies, respectively). Light grey indicates tissues with no expression data or for which we encountered no peer-reviewed reports of infection.
(TIF)

S3 Fig The variation within and between samples for all significantly variable transcripts of interest from Fig 3. The y axes show the number of transcripts per kilobase million (TPM) generated deterministically from estimated counts pseudo-aligned by kallisto, and the variation shown in each experiment is a proxy for technical replicates from 1000 bootstrap samples. The x axes group the experimental samples by condition.
(TIF)

S4 Fig I_CpG_ and nucleotide proportions for seven coronaviruses with complete, untrimmed, genomes and host information. Panel a) shows that SARS-CoV-2 has the least I_CpG_ in comparison to other coronaviruses from their natural hosts. Panels b), c) and d) respectively show that the P_U_ negatively correlates with P_C_ but not with P_A_ or P_G_; P_U_ is highest among Bovine CoV, Canine CoV (CRCoV), and Porcine HEV (HEV) but lowest among Murine MHV (MHV) and human coronaviruses. Each panel includes 2666 SARS-CoV-2 genomes, 403 MERS genomes, 134 SARS-CoV genomes, 20 Bovine CoV genomes, two CRCoV genomes, 26 MHV genomes, and ten HEV genomes.
(TIF)

S5 Fig Variations of a) ICpG and b) PU among trimmed genomes in six coronaviruses. Canine CoV (CRCoV) was omitted because only two genomes had been identified. The sample size for each category is denoted by ‘n’. Median ICpG is represented by a white dot, black rectangles represent the interquartile range. The width of yellow regions corresponds with the frequency range of ICpG and PU values.
(TIF)

S6 Fig Median I_XpY_ shows I_CpG_ is most deficient among all 16 XY dinucleotide combinations (where X and Y are A, C, G or U) in all seven coronaviruses. Each bar value displays the median I_XpY_ calculated from 2666 SARS-CoV-2 genomes, 403 MERS genomes, 134 SARS-CoV genomes, 20 Bovine CoV genomes, two CRCoV genomes, 26 MHV genomes, and ten HEV. All genomes are complete, with ends trimmed after MAFFT alignment, and have host information.
(TIF)

S7 Fig Geographical and temporal SNP patterns in 80 complete and high quality SARS-CoV-2 strains collected in the United States. Panels respectively show mutations within the E region, M region, N region, ORF3a, ORF6, ORF7a, ORF8, ORF10, S region, and 3’ UTR, in pair-wise comparison between 79 strains and the oldest strain collected in the United States (accession MN985325, sampled 2020-01-19). N denotes the number of samples per time range.
(TIF)

S8 Fig Geographical and temporal SNP patterns in 39 complete and high quality SARS-CoV-2 strains collected in Australia. Panels respectively show mutations within the N region, M region, E region, ORF3a, ORF7a, ORF8, S region, and 3’ UTR, in pair-wise comparison between 38 strains and the oldest strain collected in Australia (accession MT450920, sampled 2020-01-25). ORF6 and ORF10 regions were omitted because there were no observed mutations in these regions. N denotes the number of samples per time range.
(TIF)

S9 Fig Geographical and temporal SNP patterns in 34 complete and high quality SARS-CoV-2 strains collected in China. Panels respectively show mutations within the M region, N region, ORF3a, ORF8, S region, and 3’ UTR, in pair-wise comparison between 33 strains and the oldest strain collected in the China (accession MN908947, sampled 2019-12-31). E, ORF6, ORF7a, and ORF10 regions were omitted because there were no observed mutations in these regions. N denotes the number of samples per time range.
(TIF)

S10 Fig Site-specific C to U mutations when the reference SARS-CoV-2 strain Wuhan-Hu-1 (accession MN908947, sampled 2019-12-31) was compared to 98 SARS-CoV-2 genomes collected worldwide with unique collection dates. A) The locations of 98 unique sites having C to U mutations in the Wuhan-Hu-1 genome with annotated viral regions. B) The total count number of unique C to U mutations sites per 1000 nucleotide bases in the Wuhan-Hu-1 genome.
(TIF)

S11 Fig The relative mRNA expressions of *AID*, *ADAR*, and *APOBEC1* in 27 human tissues. “Proportions of expression” on the y axis is measured by tissue median TPM/sum tissue median TPM for each gene. Human tissue-specific mRNA expressions, in median TPM values, were retrieved from all RNA-Seq datasets available in the GTEx Portal.
(TIF)

S1 Table The summed number of C to U mutations and other mutations (non-C to U) that were observed at each viral region when the reference SARS-CoV-2 strain Wuhan-Hu-1 (accession MN908947) was compared to 98 SARS-CoV-2 genomes collected worldwide with unique collection dates. (PDF)

S1 File File S1 is the dataset containing reference compilation of virus regular habitats, tissue total RNA AVP mRNA expressions, and LoM AVP mRNA expressions. (XLSX)

S2 File File S2 is the dataset containing nucleotide and di-nucleotide frequencies in trimmed viral genomes. (XLSX)

S3 File File S3 is the dataset containing nucleotide and di-nucleotide frequencies in whole, un-trimmed, viral genomes. (XLSX)

S4 File File S4 is the dataset contains the global, local, temporal, and geographical SNP patterns in SARS-CoV-2 genomes. (XLSX)

S5 File File S5 is the dataset that contains the local CpG dinucleotide frequencies in a sample of 99 SARS-CoV-2 genomes. (XLSX)

S6 File File S6 is the dataset that contains all sequence and structural context analyses for C to U mutations. (XLSX)
