# Supplementary figures and images for: Coronavirus genomes carry the signatures of their habitats
Source: PLoS One. 2020 Dec 22;15(12):e0244025. doi: 10.1371/journal.pone.0244025 (PMC7755226; doi:10.1371/journal.pone.0244025)

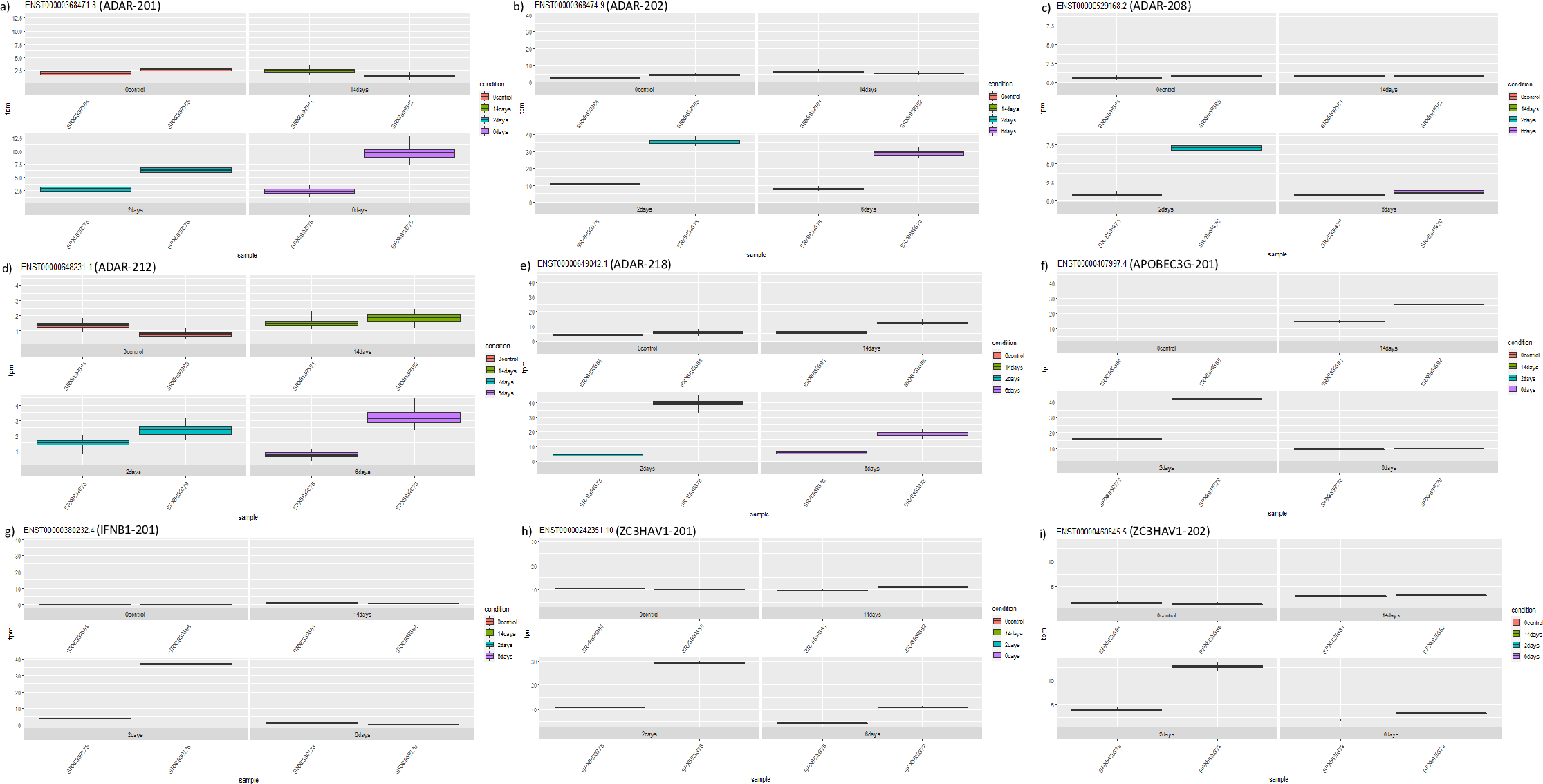

Supplement: S3 Fig — The y axes show the number of transcripts per kilobase million (TPM) generated deterministically from estimated counts pseudo-aligned by kallisto, and the variation shown in each experiment is a proxy for technical replicates from 1000 bootstrap samples. The x axes group the experimental samples by condition. (TIF) [file pone.0244025.s003.tif]

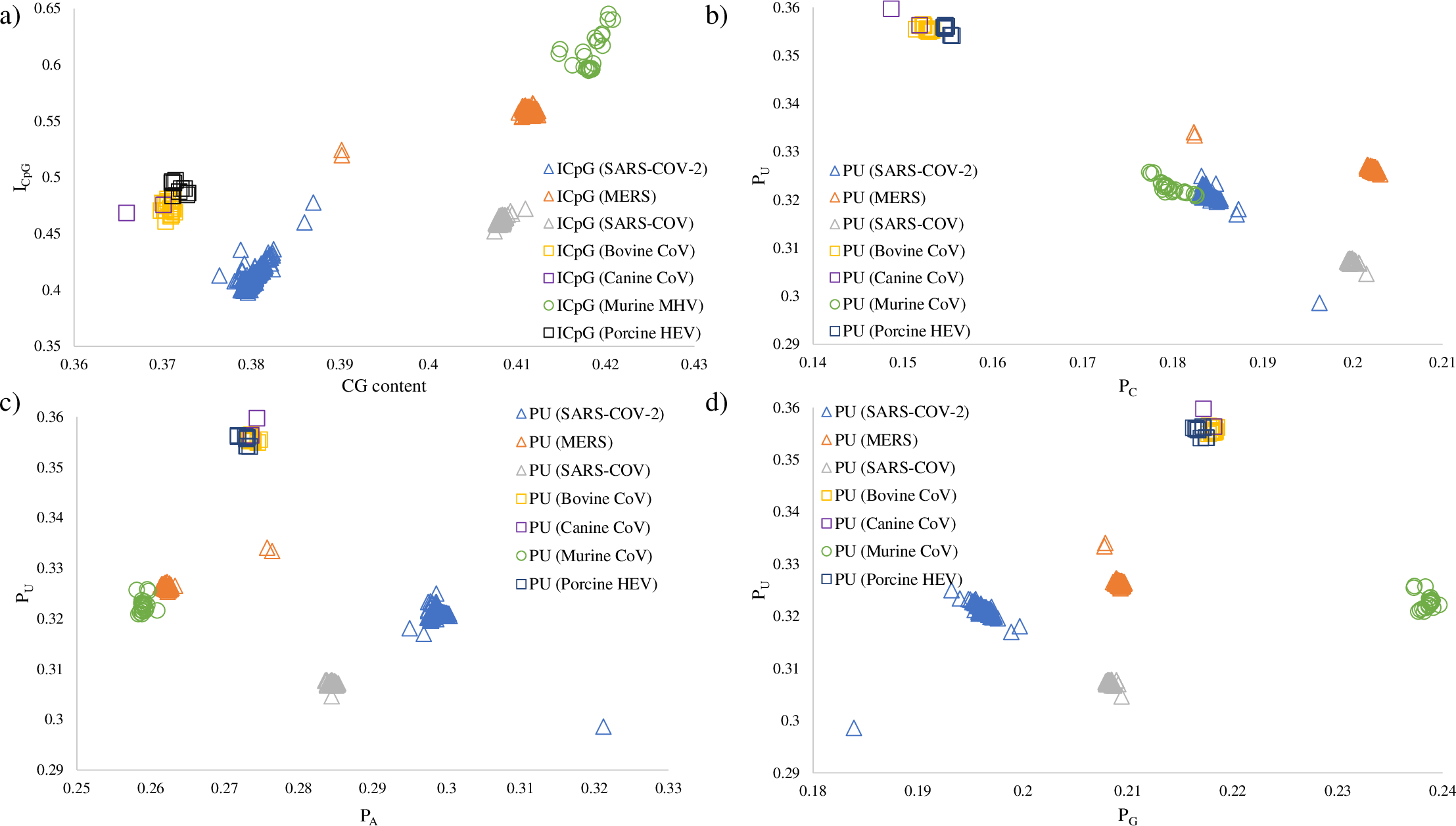

Supplement: S4 Fig — Panel a) shows that SARS-CoV-2 has the least ICpG in comparison to other coronaviruses from their natural hosts. Panels b), c) and d) respectively show that the PU negatively correlates with PC but not with PA or PG; PU is highest among Bovine CoV, Canine CoV (CRCoV), and Porcine HEV (HEV) but lowest among Murine MHV (MHV) and human coronaviruses. Each panel includes 2666 SARS-CoV-2 genomes, 403 MERS genomes, 134 SARS-CoV genomes, 20 Bovine CoV genomes, two CRCoV genomes, 26 MHV genomes, and ten HEV genomes. (TIF) [file pone.0244025.s004.tif]

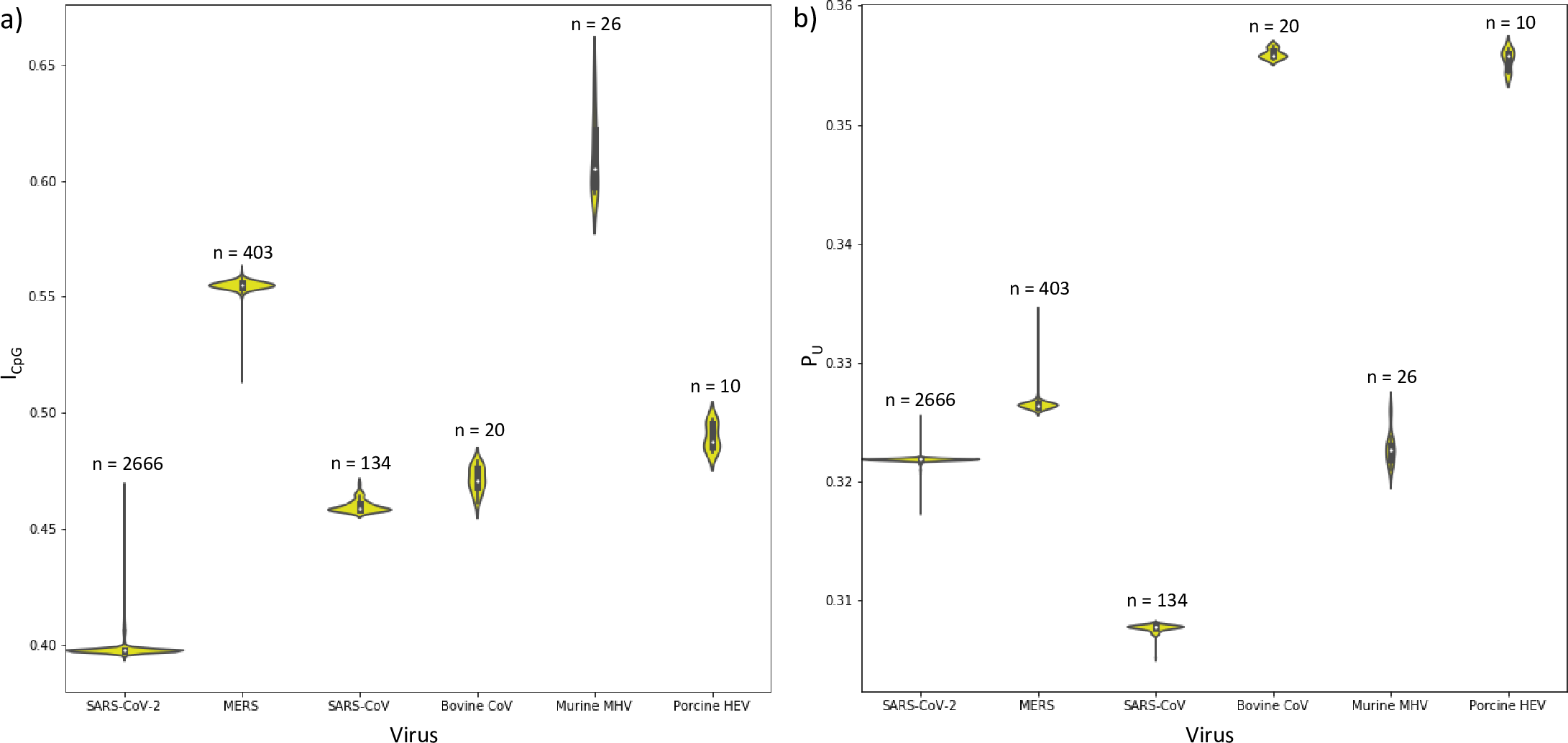

Supplement: S5 Fig — Variations of a) ICpG and b) PU among trimmed genomes in six coronaviruses. Canine CoV (CRCoV) was omitted because only two genomes had been identified. The sample size for each category is denoted by ‘n’. Median ICpG is represented by a white dot, black rectangles represent the interquartile range. The width of yellow regions corresponds with the frequency range of ICpG and PU values. (TIF) [file pone.0244025.s005.tif]

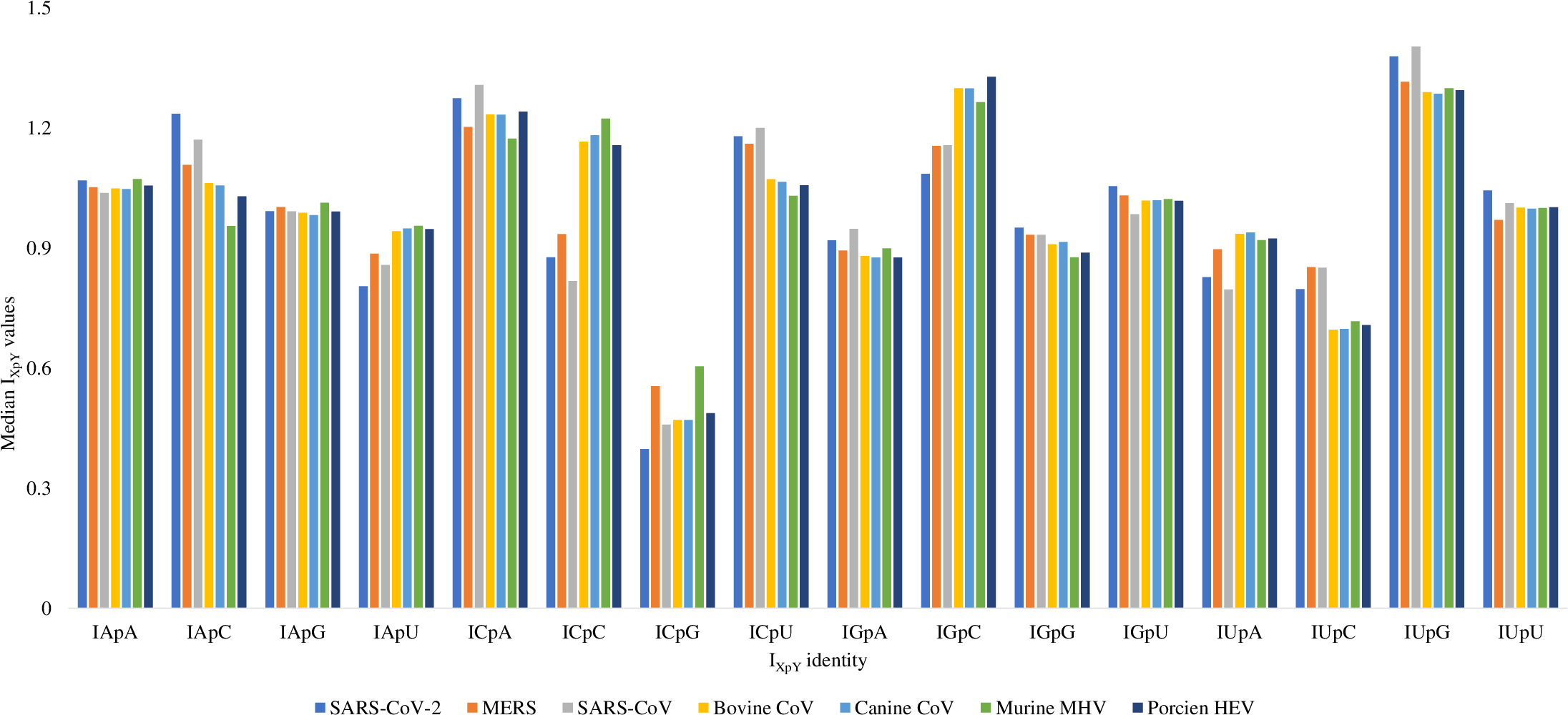

Supplement: S6 Fig — Each bar value displays the median IXpY calculated from 2666 SARS-CoV-2 genomes, 403 MERS genomes, 134 SARS-CoV genomes, 20 Bovine CoV genomes, two CRCoV genomes, 26 MHV genomes, and ten HEV. All genomes are complete, with ends trimmed after MAFFT alignment, and have host information. (TIF) [file pone.0244025.s006.tif]

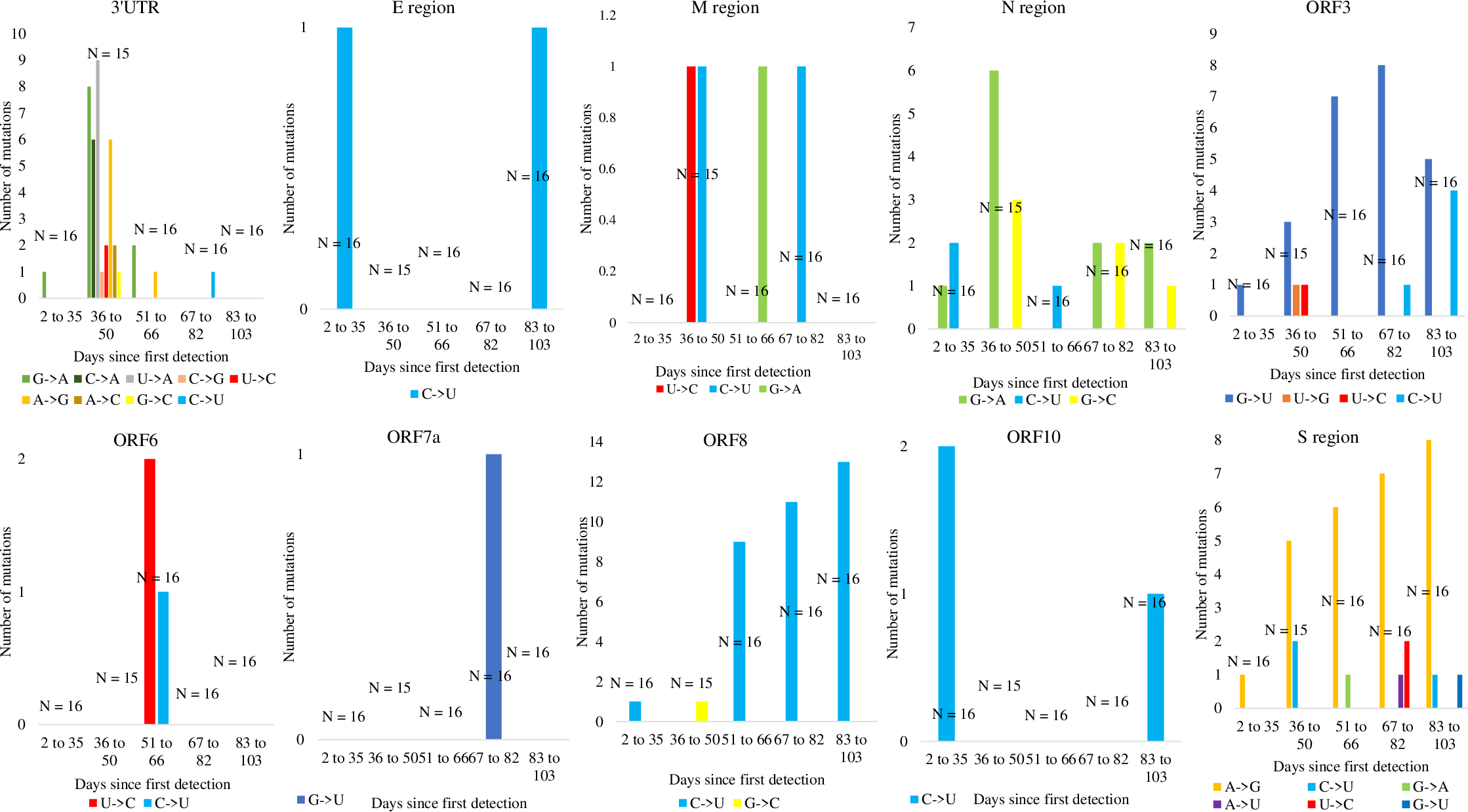

Supplement: S7 Fig — Panels respectively show mutations within the E region, M region, N region, ORF3a, ORF6, ORF7a, ORF8, ORF10, S region, and 3’ UTR, in pair-wise comparison between 79 strains and the oldest strain collected in the United States (accession MN985325, sampled 2020-01-19). N denotes the number of samples per time range. (TIF) [file pone.0244025.s007.tif]

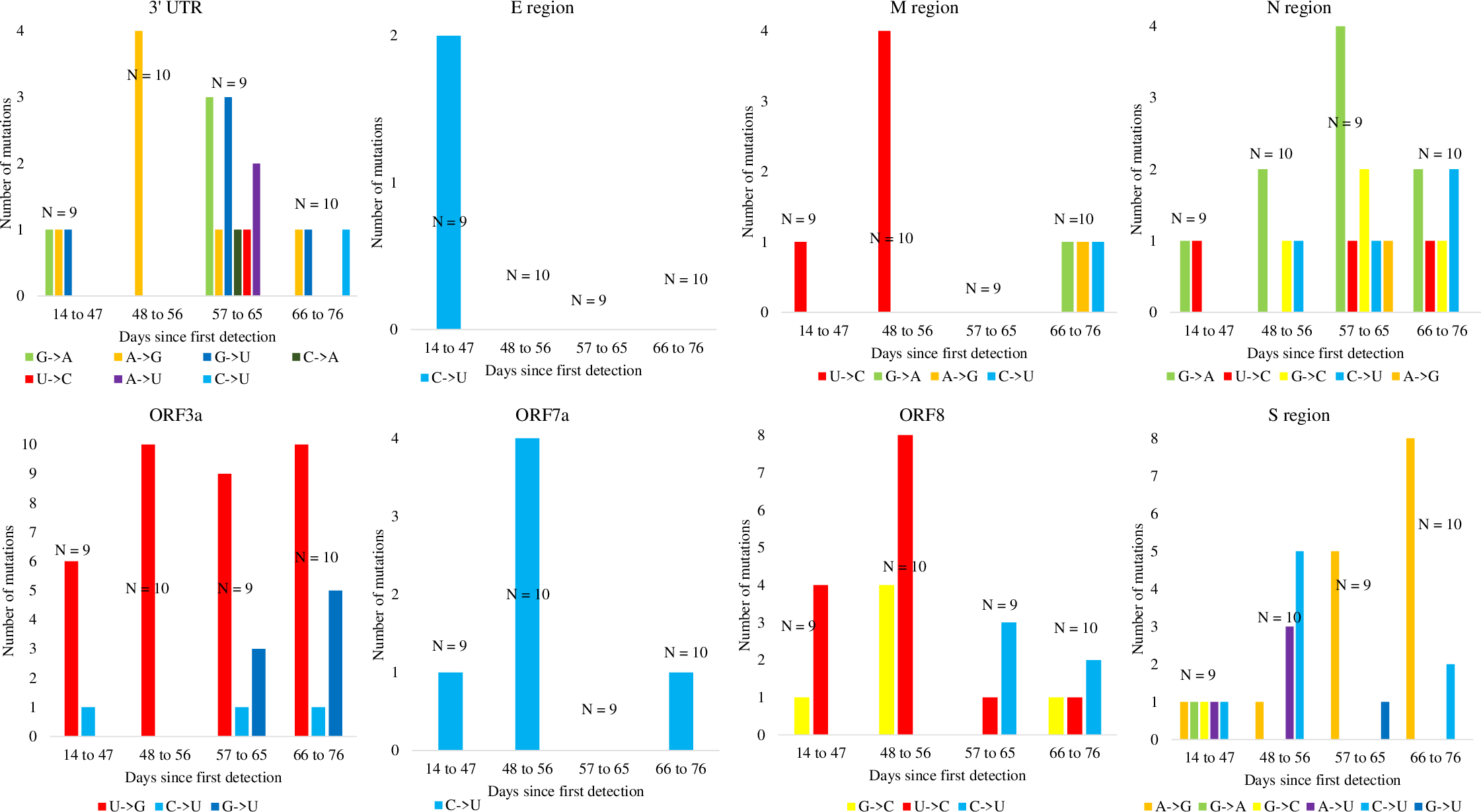

Supplement: S8 Fig — Panels respectively show mutations within the N region, M region, E region, ORF3a, ORF7a, ORF8, S region, and 3’ UTR, in pair-wise comparison between 38 strains and the oldest strain collected in Australia (accession MT450920, sampled 2020-01-25). ORF6 and ORF10 regions were omitted because there were no observed mutations in these regions. N denotes the number of samples per time range. (TIF) [file pone.0244025.s008.tif]

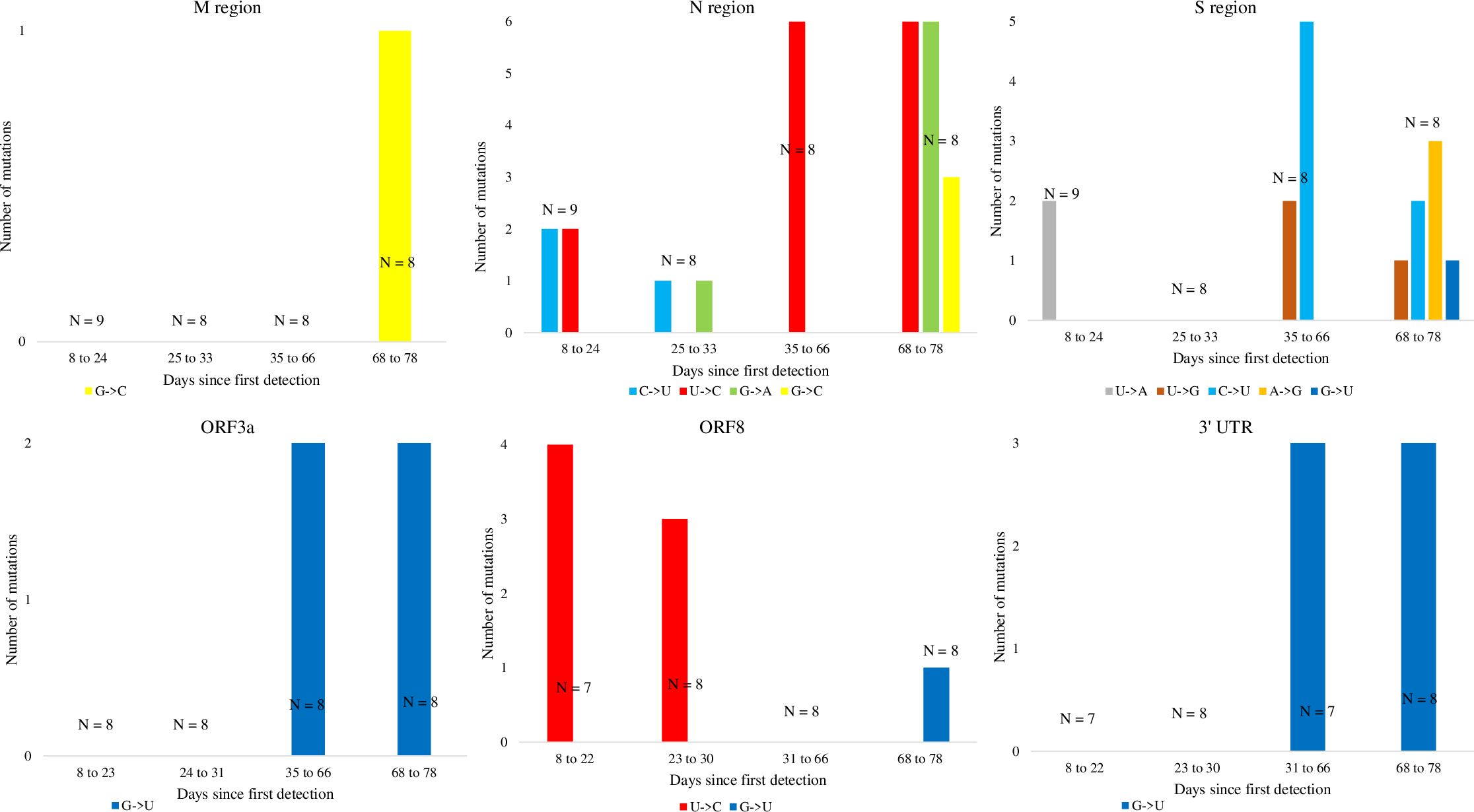

Supplement: S9 Fig — Panels respectively show mutations within the M region, N region, ORF3a, ORF8, S region, and 3’ UTR, in pair-wise comparison between 33 strains and the oldest strain collected in the China (accession MN908947, sampled 2019-12-31). E, ORF6, ORF7a, and ORF10 regions were omitted because there were no observed mutations in these regions. N denotes the number of samples per time range. (TIF) [file pone.0244025.s009.tif]

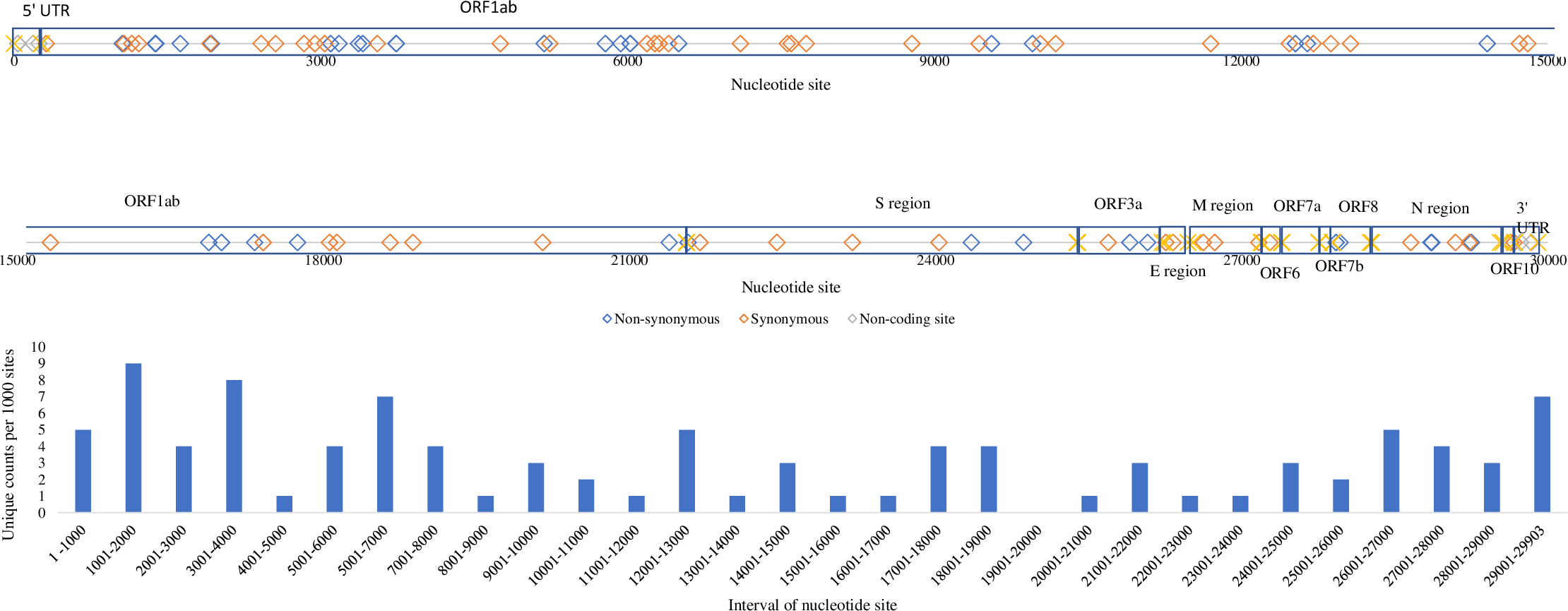

Supplement: S10 Fig — A) The locations of 98 unique sites having C to U mutations in the Wuhan-Hu-1 genome with annotated viral regions. B) The total count number of unique C to U mutations sites per 1000 nucleotide bases in the Wuhan-Hu-1 genome. (TIF) [file pone.0244025.s010.tif]

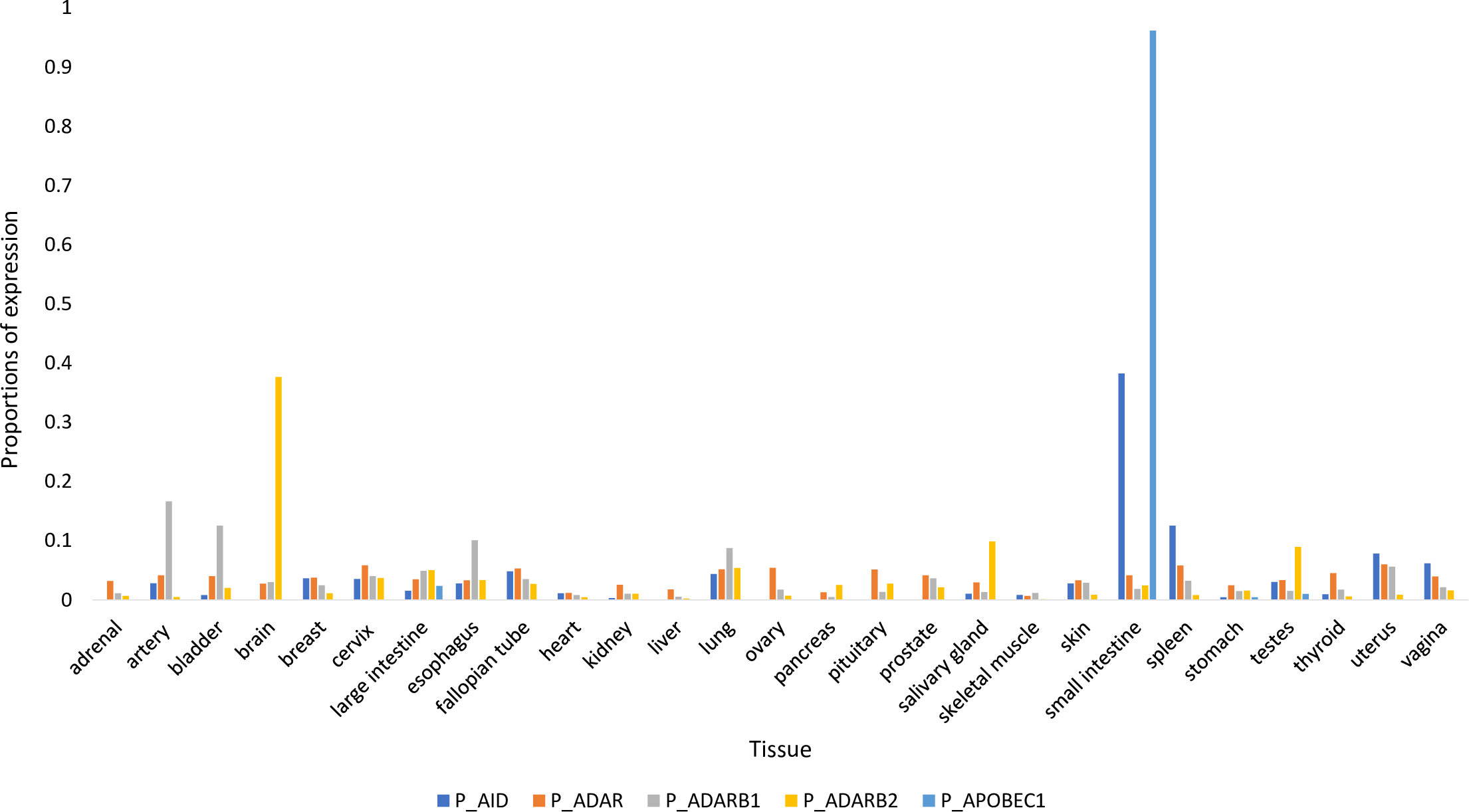

Supplement: S11 Fig — “Proportions of expression” on the y axis is measured by tissue median TPM/sum tissue median TPM for each gene. Human tissue-specific mRNA expressions, in median TPM values, were retrieved from all RNA-Seq datasets available in the GTEx Portal. (TIF) [file pone.0244025.s011.tif]
