# Supplementary material for: Coronavirus genomes carry the signatures of their habitats
Source: PLoS One. 2020 Dec 22;15(12):e0244025. doi: 10.1371/journal.pone.0244025 (PMC7755226; doi:10.1371/journal.pone.0244025)
Supplement: S1 Table — (PDF) [file pone.0244025.s012.pdf]

S1 Table. The summed number of C to U mutations and other mutations (non-C to U) that were observed at each viral region when the reference SARS-CoV-2 strain Wuhan-Hu-1 (accession MN908947) was compared to 98 SARS-CoV-2 genomes collected worldwide with unique collection dates.

Viral region	Sequence length	Synonymous (C to U)	Non-Synonymous (C to U)	Non-coding (C to U)	Synonymous (non C to U)	Non-synonymous (non C to U)	Non-coding (non-C to U)
**5' UTR**	265	-	-	35	-	-	0
**ORF1**	21291	124	66	-	26	75	-
**S**	3819	3	3	-	5	44	-
**ORF3a**	828	2	2	-	0	34	-
**E**	225	2	0	-	0	0	-
**M**	666	5	0	-	0	1	-
**ORF6**	186	1	0	-	2	0	-
**ORF7a**	363	0	0	-	0	1	-
**ORF8**	363	0	6	-	0	0	-
**N**	1257	4	5	-	3	20	-
**ORF10**	114	3	0	-	0	0	-
**3' UTR**	229	-	-	1	-	-	4
**Total**	29112	144	82	36	36	175	4
